# Oral language interventions can improve language outcomes in children with neurodevelopmental disorders: A systematic review and meta‐analysis

**DOI:** 10.1002/cl2.1368

**Published:** 2023-11-27

**Authors:** Enrica Donolato, Enrico Toffalini, Kristin Rogde, Anders Nordahl‐Hansen, Arne Lervåg, Courtenay Norbury, Monica Melby‐Lervåg

**Affiliations:** ^1^ CREATE University of Oslo Oslo Norway; ^2^ Department of General Psychology University of Padova Padova Italy; ^3^ Department of Special Needs Education University of Oslo Oslo Norway; ^4^ Department of Education, ICT and Learning Østfold University College Halden Norway; ^5^ Division of Psychology & Language Sciences University College London London UK

## Abstract

**Background:**

Young people who fail to develop language as expected face significant challenges in all aspects of life. Unfortunately, language disorders are common, either as a distinct condition (e.g., Developmental Language Disorder) or as a part of another neurodevelopmental condition (e.g., autism). Finding ways to attenuate language problems through intervention has the potential to yield great benefits not only for the individual but also for society as a whole.

**Objectives:**

This meta‐analytic review examined the effect of oral language interventions for children with neurodevelopmental disorders.

**Search Methods:**

The last electronic search was conducted in April 2022.

**Selection Criteria:**

Intervention studies had to target language skills for children from 2 to 18 years of age with Developmental Language Disorder, autism, intellectual disability, Down syndrome, Fragile X syndrome, and Williams syndrome in randomised controlled trials or quasi‐experimental designs. Control groups had to include business‐as‐usual, waiting list, passive or active conditions. However, we excluded studies in which the active control group received a different type, delivery, or dosage of another language intervention. Eligible interventions implemented explicit and structured activities (i.e., explicit instruction of vocabulary, narrative structure or grammatical rules) and/or implicit and broad activities (i.e., shared book reading, general language stimulation). The intervention studies had to assess language skills in receptive and/or expressive modalities.

**Data Collection and Analysis:**

The search provided 8195 records after deduplication. Records were screened by title and abstract, leading to full‐text examinations of 448 records. We performed Correlated and Hierarchical Effects models and ran a retrospective power analysis via simulation. Publication bias was assessed via *p*‐curve and precision‐effect estimate.

**Main Results:**

We examined 38 studies, with 46 group comparisons and 108 effects comparing pre‐/post‐tests and eight studies, with 12 group comparisons and 21 effects at follow‐up. The results showed a mean effect size of *d* = 0.27 at the post‐test and *d* = 0.18 at follow‐up. However, there was evidence of publication bias and overestimation of the mean effects. Effects from the meta‐analysis were significantly related to these elements: (1) receptive vocabulary and omnibus receptive measures showed smaller effect sizes relative to expressive vocabulary, grammar, expressive and receptive discourse, and omnibus expressive tests; and (2) the length of the intervention, where longer sessions conducted over a longer period of time were more beneficial than brief sessions and short‐term interventions. Neither moderators concerning participants’ characteristics (children's diagnosis, diagnostic status, age, sex, and non‐verbal cognitive ability and severity of language impairment), nor those regarding of the treatment components and implementation of the language interventions (intervention content, setting, delivery agent, session structure of the intervention or total number of sessions) reached significance. The same occurred to indicators of study quality. The risk of bias assessment showed that reporting quality for the studies examined in the review was poor.

**Authors’ Conclusions:**

In sum, the current evidence base is promising but inconclusive. Pre‐registration and replication of more robust and adequately powered trials, which include a wider range of diagnostic conditions, together with more long‐term follow‐up comparisons, are needed to drive evidence‐based practice and policy.

## 1 PLAIN LANGUAGE SUMMARY

### 1.1 Language interventions can improve speaking skills in children with neurodevelopmental disorders

This meta‐analytic review demonstrates that language interventions can improve oral language in children with neurodevelopmental disorders. However, this result should be interpreted with caution because of poor reporting in many studies and publication bias: selective reporting of research results in this field, based on their positive findings.

### 1.2 What is this review about?

We assessed interventions that target language skills in children with neurodevelopmental disorders. The interventions had to use techniques ranging from explicit and structured activities (explicit instruction of vocabulary, narrative structure or grammatical rules) to implicit and broad activities (shared book reading, general language stimulation). We examined whether the interventions had an impact on language in general, or on more specific aspects of language in both receptive and expressive modalities.

**What is the aim of this review?**

This review examines 42 publications reporting on the effects of oral language interventions in children with neurodevelopmental disorders.

### 1.3 What studies are included?

We evaluated the effects of oral language interventions in children with neurodevelopmental disorders at post‐test (38 studies with 46 group comparisons and 108 effects) and at follow‐up (eight studies with 12 group comparisons and 21 effects). Most of the interventions targeted children with language disorders and children with autism, and only a few involved children with Down syndrome, Fragile X syndrome, or mixed samples. The studies spanned the period 1993 to 2022 and were mostly carried out in the USA and UK.

### 1.4 Do oral language interventions attenuate language problems in children across different neurodevelopmental disorders?

Oral language interventions yield moderate effects on language skills in favour of the treatment groups at post‐test, and smaller effects at follow‐up. Importantly, the quality of evidence and risk of bias are unclear because of poor reporting of critical aspects of the study design, such as recruitment and randomisation. Overall, the analyses indicate potential publication bias, with small positive studies tending to yield larger treatment effects.

### 1.5 What factors affect how well oral language interventions work?

From pre‐ to post‐test, participants’ characteristics and treatment components and implementation of the language interventions were not significant moderators. (Participants’ characteristics: children's diagnosis, diagnostic status, age, sex, and non‐verbal cognitive ability and severity of language impairment. Treatment components and implementation: intervention content, setting, delivery agent, session structure of the intervention or total number of sessions.) However, smaller effects emerged for receptive vocabulary and multi‐component receptive measures compared to expressive vocabulary, grammar, expressive and receptive discourse, and multi‐component expressive tests. Longer sessions conducted over a longer period were more beneficial than brief sessions and short‐term interventions.

### 1.6 What do the findings of this review mean?

The current evidence base is promising but inconclusive. To drive evidence‐based practice and policy, we need pre‐registration and replication of more robust and adequately powered trials. Studies should include a wider range of diagnostic conditions. Further research should also report on long‐term follow‐up.

### 1.7 How up‐to‐date is this review?

The review authors searched for studies up to April 2022.

## 2 BACKGROUND

### 2.1 The problem, condition or issue

Oral language is an important skill that most children master during their development. Language content, language structure and functional use (pragmatics) all lay the foundation for other key cognitive and social achievements (Stothard et al., 1998) and reading comprehension (Duff et al., 2015; Lepola et al., 2016; Nation & Norbury, 2005). For instance, language is fundamental for children to communicate needs, participate in social interactions, engage in play and participate actively in society (Snow, 2021). However, a sizeable number of children experience language problems, either as an independent condition or in combination with other learning or developmental disorders. For these children, it is critical to receive support and interventions that might prevent the language problems from having detrimental consequences on their life course and functioning. Oral language interventions may improve oral language competencies in different neurodevelopmental disorders that are characterised by varying degrees of language deficit.

Oral language is a multifaceted system that comprises vocabulary (semantics), grammar (syntax and morphology) and discourse processing (pragmatics) in both the expressive (language production) and receptive (language comprehension) domains (Lervåg et al., 2018). In the course of language development, the receptive and expressive language domains go hand in hand, although comprehension of language starts to develop slightly earlier compared with expressive skills (Hulme & Snowling, 2013). The development of vocabulary is a core ingredient in language development (Marchman & Fernald, 2008; Melby‐Lervåg & Lervåg, 2014), and measures of expressive and receptive vocabulary are widely used in interventions that include children with neurodevelopmental disorders. In addition to vocabulary development, oral language skills encompass grammar, which includes morphology (word formation) and syntax (sentence formation), as well as narrative and discourse development (Hulme & Snowling, 2014).

It is important to note that language disorder is not a low‐incidence condition, language deficits are common and thus frequently encountered in community child development clinics (O'Hare, 2013). As for prevalence, Black et al. (2015) reported on data from the National Health Interview Survey in the US; in their findings, 7.7% of parents reported that their children aged 3–17 years old had experienced language problems in the past year. A recent population‐based survey conducted in England estimated the prevalence of children with language problems from a currently unknown cause to be 7.58% (consistent with previous epidemiological studies of “specific language impairment” conducted in North America; Beitchman et al., 1986; Tomblin et al., 1997), whereas 2.34% of children had language deficits as part of another condition (Norbury et al., 2016).

Those who have language problems as a part of another condition have more severe language deficits and are more likely to have co‐occurring non‐verbal IQ deficits and social, emotional and behavioural problems (Norbury et al., 2016). They were also more likely to be receiving special education support, although not necessarily more speech‐language therapy. Norbury et al. (2015) also demonstrated that teacher‐rated language problems were the single best predictor of academic success during the first year of school. A large portion of these children belong under the umbrella of neurodevelopmental disorders (Bishop & Rutter, 2008; D'Souza & Karmiloff‐Smith, 2017). Some of these diagnoses have a known genetic or acquired aetiology, such as Down syndrome, Williams syndrome or Fragile X syndrome, whereas other diagnoses, such as language disorder, intellectual disability and autism, have multifactorial aetiologies that are less well understood (Thapar & Rutter, 2015). However, one common characteristic of neurodevelopmental disorders is that affected children often display language difficulties, and thus, require systematic support and interventions that target oral language.

Thus, language disorder is a rather common problem, both as a distinct diagnostic condition (DLD) and as a part of more pervasive neurodevelopmental conditions. Language disorder can have a large impact on an individual's life course and substantially increases risk for adverse outcomes in education, employment, social well‐being and mental health (Dubois et al., 2020), representing significant costs to society (Cronin et al., 2020). While intervention research and services have traditionally been developed to address specific diagnostic groups, there are potentially common learning strategies that could apply across diagnostic boundaries. Finding efficient interventions and ways to support those who are affected might have a positive impact not only for the individual but also for society. Here we will address this issue and summarise studies that have used different kinds of oral language intervention with different clinical populations.

#### 2.1.1 The value of a transdiagnostic approach to language intervention

The CATALISE consortium (Bishop et al., 2016, 2017) highlighted clinical assumptions that children with different neurodevelopmental disorders may require different therapeutic approaches or that children with non‐verbal cognitive deficits may not benefit from oral language interventions to the same extent that their cognitively able peers do. However, there is limited evidence directly comparing intervention effects across neurodevelopmental disorders on which to make this judgement. Importantly, the language trajectories for children with neurodevelopmental disorders are complex, and there are small to substantial variations in language acquisition both within and across diagnostic groups. In addition, many studies show that there can be pervasive deficits within different subcomponents of language for these children, necessitating assessment across the subcomponents of oral language (Norbury & Paul, 2015). However, assessing language skills in young children in a reliable and valid way is challenging.

A transdiagnostic method that compares children with different neurodevelopmental disorders enables the investigation of unique versus similar approaches. Several primary studies of language profiles have included direct comparisons of different neurodevelopmental disorders. For instance, one study compared children with Williams syndrome and children with “specific language impairment” and reported distinct patterns of syntactic binding (Ring & Clahsen, 2005). Differences in language profiles have also been reported between children with Fragile X syndrome and Down syndrome; in this case, autism symptom severity was associated with language differences between these two groups (Martin et al., 2013; Price et al., 2007). At the same time, children with autism, Down syndrome, Williams syndrome, Fragile X syndrome or an intellectual disability all display some degree of language deficit (Abbeduto et al., 2016; Rice et al., 2005). Therefore, another reason to focus on children with different neurodevelopmental disorders is that there are considerable overlaps in the severity and pattern of language deficit and/or language strengths (Gibson et al., 2013), shared aetiological risk factors (Valenti et al., 2014) and commonalities in cognitive profiles (Raitano Lee et al., 2016). In addition, there are high rates of comorbidity among these groups of children (Abbeduto et al., 2016; American Psychiatric Association [APA], 2013), and diagnostic categories are not as distinct as once thought (Thapar & Rutter, 2015). Nevertheless, whether similar oral language interventions provide similar levels of benefit for children with different neurodevelopmental disorders, or whether different interventions are needed, remains an unanswered question.

### 2.2 Description of the condition

#### 2.2.1 Neurodevelopmental disorders included in the review

In this review, we focus on *Developmental Language Disorder* and associated *differentiating conditions* identified by the CATALISE consortium (Bishop et al., 2017). These are biomedical conditions in which language impairment is one of a complex set of symptoms, as in autism or intellectual disabilities. These are distinguished from *co‐occurring conditions*, such as learning disorders and attention deficit hyperactivity disorder (ADHD), in which language difficulties occur at higher than expected rates, but are not always present or characteristic of these conditions.

##### Multi‐factorial disorders without known genetic aetiology

###### Language disorder

Language disorder refers to deficits in receptive or expressive language in vocabulary, sentence structure or discourse (APA, 2013). Depending on the diagnostic criteria and cut‐offs, the prevalence rates vary greatly, with reports ranging from 2% (Weindrich et al., 2000) to 31% (Jessup et al., 2008). Following the new *Diagnostic and Statistical Manual of Mental Disorders* (5th ed.; DSM‐5) criteria, a recent population study estimated the prevalence of children having a developmental language disorder of unknown origin to be approximately 7.58%, with an additional 2.34% occurring in the context of an existing medical diagnosis (Norbury et al., 2016).

The criteria for language disorder include problems in spoken and written communication starting early in the developmental period. Such difficulties cannot be explained by sensory impairments, such as hearing loss, motor dysfunction or another medical or neurological condition (APA, 2013). The core criteria relate to limited expressive or receptive oral language (vocabulary, grammar and discourse), and as noted by Norbury and Paul (2015), affected children are typically slow to acquire first words and first word combinations. During the course of development into the school years, vocabulary remains limited and is accompanied by varying degrees of grammatical error, immaturity and errors in language production, poor narrative and discourse understanding and production, and limitations in pragmatics, especially when linguistic context is important for processing (i.e., inferencing; APA, 2013).

Notably, the debate surrounding diagnostic criteria and their terminology is ongoing (Bishop et al., 2016). Although we use the DSM‐5 terminology of language disorder in this review we also take into account studies of children where other labels are used, such as developmental language disorder, receptive language disorder and specific language impairment, to name a few (see Bishop, 2017, for a discussion and variations of terms).

###### Intellectual disability

Intellectual disability is a heterogeneous condition that affects cognitive and adaptive functioning and is associated with multiple possible causes. Prevalence estimates in the overall population are reported to be approximately 1%–3% (Moeschler & Shevell, 2014). Variations in prevalence are largely due to differences in how the term intellectual disability is defined and where the cut‐off is set for impairment is set (Bishop et al., 2016).

The defining features of intellectual disability in the DSM‐5 are as follows: (1) deficits in intellectual functions, such as reasoning, learning and abstract thinking; (2) deficits in adaptive functioning; and (3) occurrence of these deficits during the developmental period (APA, 2013). Intellectual disability is further defined through the use of specifiers based on an individual's adaptive functioning, with specifiers indicating a severity level ranging from mild to moderate, severe, and profound (APA, 2013). Individuals may change their severity level, but intellectual disability is thought to be a lifelong condition.

###### Autism

Autism is an umbrella term that encompasses conditions previously labelled as childhood autism/autistic disorder, high‐functioning autism, atypical autism, Asperger syndrome and pervasive neurodevelopmental disorder not otherwise specified (APA, 2013). Some epidemiological studies report a worldwide prevalence of approximately 50–70 per 10,000 people (Zeidan et al., 2022) for the broader definition of the autism spectrum. In some parts of the UK and the US, the prevalence has been reported to be more than 100 per 10,000 children (Baird et al., 2006; Kogan et al., 2009) and as high as 157 per 10,000 children when statistically controlling for unknown cases (Baron‐Cohen et al., 2009; Fombonne, 2009).

Two areas of functioning and behaviours make up the core diagnostic criteria of autism: one consists of restricted, repetitive behaviours and interests, and the other is related to social communication and social interaction (APA, 2013). Language is highly variable within the autism spectrum. The number of children who do not acquire functional speech is estimated to be approximately 30% (Pickles et al., 2014). Even when children with autism acquire spoken language, many have language deficits that are similar to those seen in DLD (Kjelgaard & Tager‐Flusberg, 2001). For example, Loucas et al. (2008) reported that in a sample of autistic children with IQ scores above 80, 41 children had language impairments, whereas 31 children did not. Before diagnosis, the absence of first words and sentences is the most frequently reported concern for parents (De Giacomo & Fombonne, 1998; Wetherby et al., 2004).

Pragmatics is a common area of concern in autistic language development, although some aspects of pragmatics, such as the understanding of metaphors, may be associated with broader structural aspects of language, such as vocabulary and/or grammar (Kalandadze et al., 2016). Studies conducted by Norbury and colleagues lend support to the notion that the difference between children with autism (with or without language impairments) and non‐autistic children (with or without language impairments) depends on the degree of language deficit rather than the degree of autistic traits (see, for instance, Brock et al., 2008; Norbury, 2005).

##### Syndromes with a known aetiology

###### Down syndrome

Down syndrome, or Trisomy 21, is the most common known genetic cause of intellectual disability that is not inherited. The prevalence of Down syndrome has been reported in Europe and the US to be approximately 8 per 10,000 people (Presson et al., 2013). For persons with Down syndrome, the gap between cognitive abilities and chronological age has been reported to increase in adulthood (Raitano Lee et al., 2016). A meta‐analysis indicated that individuals with Down syndrome show slow, positive rates of change compared with what is expected in typically developing children (Patterson et al., 2013). Since delays and deficits in language are reported from early onset to adulthood, language interventions for this group are of particular importance (Martin et al., 2009).

Children with Down syndrome often score significantly lower than typically developing children on measures of expressive language (Finestack et al., 2013; Næss et al., 2011). For receptive vocabulary, studies have reported mixed findings. Some studies indicate a clear challenge in expressive language relative to receptive language (i.e., Glenn & Cunningham, 2005; Laws & Bishop, 2003). Further, in a systematic review on language skills in children with Down syndrome, Næss et al. (2011) reported that receptive skills were not statistically significantly different compared with those of typically developing children with the same non‐verbal mental age. However, other studies comparing children with Down syndrome to other mental age–matched groups report difficulties in receptive language (Hick et al., 2005; Roberts et al., 2007). In addition, deficits in syntactic structure and complexity are quite common (Martin et al., 2009). However, there are large within‐syndrome variations (Abbeduto et al., 2016), and some of the differences and inconsistencies reported in the language domain may be due to variations in assessment procedures used in the studies, hearing loss or variations in cognitive status across studies (Martin et al., 2009).

###### Williams syndrome

Williams syndrome is a rare multi‐system disorder caused by deletion of the Williams‐Beuren syndrome chromosome region (Pober, 2010), and it has a reported prevalence of approximately 1 in 7500 people (Strømme et al., 2002). Early onset developmental delays are typical for children with Williams syndrome. However, clinical diagnostic criteria are usually not as useful for the accurate diagnosis of Williams syndrome compared with laboratory testing (Pober, 2010). For children with this syndrome, medical conditions apply to a much larger degree compared with typically developing children (Morris, 2010). The cognitive profiles of this group are generally in the mild to moderate range for overall IQ, but there is variation in the range of approximate IQ, with scores between 40 and 100 (Martens et al., 2008). The neurocognitive profile of Williams syndrome is complex, involving relative strengths in aspects of oral language and profound weaknesses in visuospatial cognition (Mervis & John, 2010).

The discrepancy in verbal and non‐verbal skills in the Williams syndrome profile has led some to conclude that language is surprisingly preserved in this condition (Karmiloff‐Smith, 2007). However, this strength is *relative to* other areas of functioning and not necessarily within the range found in typically developing children of a similar age (Bellugi et al., 2000; Karmiloff‐Smith et al., 1997). Thus, there is a need for information on language interventions for children with Williams syndrome, especially considering that this has received little focus since their language competencies may have been overstated (D'Souza & Karmiloff‐Smith, 2017).

###### Fragile X syndrome

Fragile X syndrome is the most common genetic cause of inherited intellectual disability. Prevalence estimates for Fragile X syndrome are approximately 1 in 5500 for males (Macpherson & Murray, 2016) and approximately 1 in 8000 for females. However, prevalence estimates vary considerably, especially with advances in genetic testing (Hunter et al., 2014). Co‐occurrence with autism is high in children with Fragile X syndrome, with up to 50% scoring above cut‐offs on diagnostic tests for autism (Hall et al., 2008).

Early language milestones are delayed relative to those in typically developing children, and this difference is especially apparent for boys with Fragile X syndrome. The extent and nature of persistent language deficits are unclear because of mixed results from studies using different methodologies and measures. One reason for the imprecision in estimating language competence may be anxiety in the context of testing that these children can experience (Cornish et al., 2004). However, available evidence indicates impairments in language in children with Fragile X syndrome that include both structural and pragmatic aspects of language, particularly vocabulary (Klusek et al., 2014; Kover et al., 2015; Martin et al., 2013).

### 2.3 Description of the intervention

#### 2.3.1 Theoretical approaches to language intervention

Our starting premise is that the language impairments characteristic of neurodevelopmental disorders arise from a complex interplay of genetic and environmental risk factors and chance events (Mitchell, 2018). These risk factors do not affect language directly; instead, they affect the development of brain structure and function in ways that are non‐optimal for learning language. In addition, language acquisition is typically an interactive process in which children play an active role. Thus, because of the nature of many neurodevelopmental disorders, the quantity and quality of language input may be disrupted. Therefore, children with language disorders may require additional language input, more exposures to the same input to achieve the same level of learning relative to peers, and/or input that is structured in such a way that it is easier to learn.

**Figure 1:**
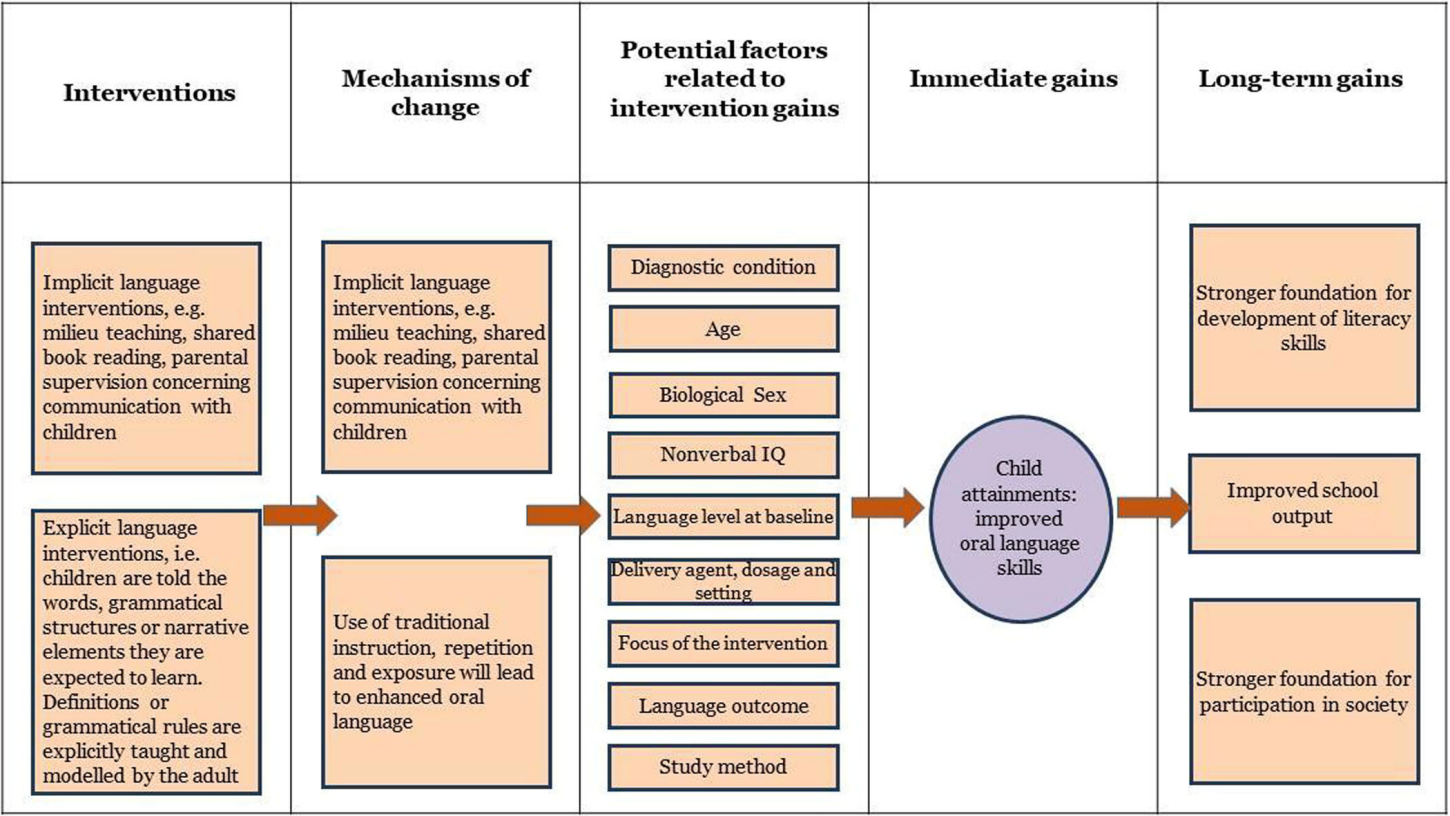
Theory of change model for the intervention.

Figure 1 shows a theory of change model for the language interventions. As shown in the figure, interventions fall along a continuum distinguished by the extent to which language instruction is explicit or language learning is implicit (i.e., the learner is not aware of the goals of the intervention; Baron & Arbel, 2022). Implicit, or incidental, approaches are grounded in developmental constructive theories of language acquisition through meaningful interactions with the child. These interventions, such as Hanen or Pediatric Autism Communication Therapy (PACT), seek to coach parents on how to optimise their language input to align with the child's attentional focus and how to identify and interpret child behaviours as potentially communicative; thus, they focus on parent‐child interactions.

Milieu therapy techniques that are implemented during child‐led play may also be included here. In this approach, interlocutors again follow the child's lead, map language onto the child's attentional focus, recast child utterances to provide more accurate language models and give the child repeated exposures to new words and language structures. Shared or dialogic book reading may be a more structured form of this approach, but this technique uses the same principles of interactive engagement with the child in a developmentally appropriate task, in which key words or structures are highlighted by the adult as they naturally occur within a meaningful story context. A common feature across the different tasks is that the children are unaware of the learning targets and may even be unaware that they are taking part in an intervention. Instead, the environment is engineered to make the desired targets more salient, but learning is incidental and includes increasing the number of exposures to language targets in naturalistic ways.

The second approach aims to circumvent underlying learning deficits via explicit instruction. This approach is more common in older children, and while targets tend to follow a normative developmental sequence, they may also reflect a “language curriculum” in which targets are more closely aligned to educational priorities. In these approaches, children are told the words, grammatical structures or narrative elements they are expected to learn. Definitions or grammatical rules are explicitly taught and modelled by the adult, followed by multiple opportunities for the child to practice producing and/or understanding these structures, typically with feedback provided in fun, child‐friendly activities. At the more extreme end of this continuum of practice, correct attempts at language production may be reinforced with external rewards. Most vocabulary interventions use explicit instruction, and grammatical interventions such as “Shape Coding” (Ebbels, 2007) make use of objects, colours and/or shapes to teach rules of grammar.

These approaches, or the hybrid interventions that combine these approaches, appear in the interventions in every neurodevelopmental disorder we have included. Adaptations to the delivery of these approaches may vary according to the severity of the child's language impairment or co‐occurring challenges in attention, behaviour or non‐verbal cognitive abilities. Typical adaptations aim to increase attentional focus and time on task; they may include visual prompts/timetables; use of rewards; changes to the frequency, intensity or duration of therapy sessions; and provision of the intervention individually or in small groups.

### 2.4 How the intervention might work

#### 2.4.1 Factors related to children's characteristics that may impact the intervention's effects

##### Diagnostic condition

The neurodevelopmental disorders included in the present systematic review have many similarities in oral language profiles. These similarities mean that effective interventions for children with one type of neurodevelopmental condition may also be effective for children with other neurodevelopmental conditions. However, there are also unique cognitive and behavioural profiles that may influence both the natural course of language development and the response to an intervention. Including a range of diagnostic conditions allows for an overall impression of the impact of oral language interventions, as well as comparative analyses of effect sizes across these conditions.

##### Age

The age of the sample might also be related to the size of intervention effects. Often, we intuitively believe that early intervention is better than later intervention. However, this is an empirical question, and for instance, Gardner et al. (2019) found no support for the “earlier is better” hypothesis in outcomes of parenting programmes for child behaviour problems across the age range of 2–11 years. Based on this and other findings, Maughan and Barker (2019) argued that careful analysis assessing age variations in intervention effects across broader age ranges and in other developmental domains may provide stronger tests for the earlier is better hypothesis. Therefore, we examined sample age as a moderator.

##### Biological sex

Sex composition in the samples is also something that might explain variations in the studies. Sex differences in early language acquisition and in language disorders are common, but they are not always present. According to the US Centers for Disease Control (2021), an autism diagnosis is four times more common in boys versus girls. There is also a higher prevalence of boys with language disorders compared with girls (Norbury et al., 2017). Fragile X syndrome is the world's most common hereditary cause of developmental delay in males, and males are more affected by this condition than females (1 in 2500 males and 1 in 8000 females; Hunter et al., 2014). Down syndrome and intellectual disability also involve a higher incidence in males compared with females (McKenzie et al., 2016), whereas WS appears to affect girls and boys equally (Morris et al., 2020). Even if most neurodevelopmental disorders have a higher prevalence in boys than girls, this does not imply that language interventions have unequal effects on boys relative to girls. However, sex differences in intervention effects may be evident, and the proportion of boys in the samples may explain variation in effect sizes between studies.

##### Non‐verbal IQ

Historically, diagnostic criteria for neurodevelopmental disorders have employed inclusion and exclusion criteria that relate to whether the non‐verbal IQ is over or below certain thresholds. For instance, to be diagnosed with “specific language impairment,” non‐verbal IQ had to be within the “normal range” for age and discrepancies between verbal and non‐verbal abilities were frequently required. However, the trend in the DSM‐5 is to downplay the role of cognitive levels as measured by traditional intelligence tests and to focus more on adaptive functioning. Similarly, the CATALISE consortium rejected the use of non‐verbal ability in the absence of intellectual disability) as an exclusion criterion for DLD (Bishop et al., 2016); moreover, non‐verbal IQ does not appear to be associated with the rate of language change, at least in the primary school years (Norbury et al., 2017). Research evidence regarding the role of non‐verbal cognitive ability in response to treatment is lacking and urgently needed. Cognitive functioning remains closely intertwined with neurodevelopmental disorders and poses a key variable that may influence variance in intervention outcomes (Rice, 2016).

##### Language level at baseline

How severe language problems are at baseline might also affect the outcomes of the interventions. Here, three competing hypotheses could be outlined: First, progress could be similar across the distribution. For instance, two trials of targeted interventions for children with low language proficiency (but without a clinical diagnosis) found that baseline levels of language did not matter for the size of the intervention effects; the children made equal amounts of progress independent of language level when the intervention started (Hagen et al., 2017; West et al., 2021). Note that this pattern would mean that the children with the most severe impairments did not achieve the same level of outcome as their more able peers. Second, it could be that a child with severe language problems to begin with can make accelerated progress because only small levels of improvement might have a bigger impact on their language skills. However, one could also predict the opposite: those with very low levels of language skills to begin with might have more profound difficulties that are hard to alter with interventions, resulting in slower rates of language progress. Therefore, we conducted an exploratory analysis to determine whether response to treatment varies according to the initial severity of language impairment.

#### 2.4.2 Factors related to components and implementation of language intervention that might influence intervention effects

##### Focus of the intervention and language skills targeted

Intervention studies vary in the extent to which they target individual components of the language system (i.e., a specific focus on vocabulary or particular syntactic constructions) versus a more generalised approach to language stimulation (i.e., more naturalistic play or discourse exchanges) that targets a wide range of language structures. Since the efficacy of the intervention may vary in relation to the language skills targeted, this variable was examined.

##### Language outcome measure

Language can be measured in several ways that may also influence the size of the treatment effect. For example, parent report versus observer ratings versus direct assessment all provide valid estimates of language, but they may provide variable estimates for the same child. Standardised instruments tend to yield smaller estimates of language change relative to bespoke measures. In a similar vein, outcome measures that are more proximal to intervention targets typically report larger treatment effects compared with more distal measures (Nordahl‐Hansen et al., 2016). Measures of language comprehension generally yield lower estimates of change relative to measures of language production (Melby‐Lervåg et al., 2020; Rogde et al., 2019).

##### Settings

Considering the challenges many children with neurodevelopmental disorders may have in transferring skills taught during the intervention to other contexts, the context of delivery is especially important. The context of delivery can vary, with interventions typically implemented in preschools and kindergartens, in schools, in clinical settings, or the child's home. The setting may also determine whether the intervention is delivered on a one‐to‐one basis or in small groups, which may also moderate treatment outcomes.

##### Delivery agents

An important aspect of intervention research relates to who delivers the intervention. Delivery agents vary depending on the context; typically, parents are the delivery agents when the intervention is delivered in the home. However, in school‐based studies, interventions can be delivered by speech‐language pathologists, teachers, researchers and/or trained teaching assistants. Therefore, the professional qualifications, experience and training available to delivery agents may moderate treatment outcomes.

##### Dosage

The amount of intervention required to affect change is a topic of heated debate; therefore, it is noteworthy that little systematic research has investigated the extent to which outcomes depend on the intervention frequency, duration or intensity (Frizelle et al., 2021; Warren et al., 2007). Dosage also includes other methods of delivery, such as booster sessions to sustain an intervention effect following the initial intervention period. Dosage is an important aspect of intervention research because it is inevitably tied to time‐, resource‐ and cost‐efficiency constraints. Determining whether some neurodevelopmental disorders require different dosages to achieve the same treatment effect could usefully inform effective service planning.

### 2.5 Why it is important to do this review

We essentially have a transdiagnostic approach to education (Astle et al., 2022), but our intervention research has tended to be narrowly focused along diagnostic lines. Therefore, there is a need to map interventions across a range of neurodevelopmental conditions to gain a better understanding of what works for whom, why, and under what conditions. Further, there is an urgent need to investigate potential moderators of treatment effects, given the scarcity of evidence that such variables influence outcomes (Norbury et al., 2016). This issue is particularly relevant considering changes in diagnostic criteria for language disorders to include children with more variable cognitive profiles (Bishop et al., 2017). Finally, the review is also important because it aims to highlight areas that require replication or for which current evidence is lacking.

In the protocol for this review, Nordahl‐Hansen et al. (2019), presented previous reviews and meta‐analytic studies evaluating the effects of language interventions in children defined as having “specific” language disorders or primary speech and/or language disorders. The are several previous reviews in this area that have focused on speech‐language pathologists as the primary agent of intervention delivery (Cirrin & Gillam, 2008; Cirrin et al., 2010; Gerber et al., 2012; Law et al., 2004). There are also several meta‐analyses concerning children with autism and different kinds of language interventions that show promising effects (Hampton & Kaiser, 2016; Sandbank et al., 2020). Further, a recent systematic review of children with Down syndrome has shown that these children might also benefit from language interventions (Smith et al., 2020). As for the neurodevelopmental disorders with relatively low incidence, there have mainly been narrative systematic reviews that also consider effects from previous language intervention studies (e.g., Erickson et al., 2018, for Fragile X syndrome). However, these previous meta‐analyses and systematic reviews mainly look at one neurodevelopmental condition and exclude the others. An exception is the meta‐analysis conducted by Roberts and Kaiser (2011), which included children with “all types of language impairments” in addition to intellectual impairments and autism. However, the authors included only parent‐implemented interventions, whereas our review considers clinician‐ and educator‐led interventions that may be particularly relevant to older children. Overall, although there are many reviews of language interventions, no reviews have examined the efficacy of oral language interventions across a broad inclusion of children with neurodevelopmental disorders, evaluated in a cross‐disorder manner. Thus, the main contribution of this review is to elucidate whether there are differences in the types of interventions offered, or the responses to interventions between neurodevelopmental disorders, which can enhance our understanding of whether tailored interventions are needed for specific conditions. In addition, the present review has clinical implications and may guide clinicians, therapists, practitioners and parents in selecting optimal interventions for these children.

From a societal perspective, this systematic review can inform the development of policy and best practice for children with neurodevelopmental disorders. In addition to covering a comprehensive range of diagnostic conditions, we examined children with neurodevelopmental disorders from preschool to school years in an attempt to map not only the effect of early interventions but also the potential for language change in older children. A heightened focus on oral language interventions for school‐aged children is needed because language disorders are often persistent, while the language needs of educational curricula and social interactions increase in complexity over time (Norbury, 2015). This focus also taps into a topic of debate in practice and policy regarding the optimal age at which children may be most responsive to intervention (Norbury, 2015).

It is worth emphasising that interventions targeting language in children are plagued by a lack of rigour, especially considering the provision of a sound theoretical rationale and evidence for efficacy (Hulme & Melby‐Lervåg, 2015). Therefore, contributions to building a sounder evidence base in this field are critical and can provide information about what works, as well as uncovering what does not. The proposed review also aims to highlight areas where evidence is lacking, provide an overview of evidence quality for a range of neurodevelopmental disorders, and outline priorities for future research.

## 3 OBJECTIVES

In this systematic review, we aimed to investigate the effects of oral language interventions for children with DLD, intellectual disability, autism, Down syndrome, Williams syndrome and Fragile X syndrome. Language development is a highly frequent area of difficulty for children within these diagnostic groups, and therefore, oral language interventions are important. However, to provide better evidence‐informed practice, we need to adopt a transdiagnostic approach in which we look at effects from interventions across different disorders, considering common elements of language interventions and their effects that transcend traditional diagnostic boundaries.

The primary objective of this review is to evaluate the effect of interventions that aim to increase oral language skills in children with different neurodevelopmental disorders. The research questions addressed in this review are as follows:

- How effective are oral language interventions for children across different neurodevelopmental disorders?
- Is there evidence that oral language interventions are effective at follow‐up?
- Are treatment effects robust once publication bias is examined?
- What factors do moderate the response to treatment? The factors tested included several variables related to the following: 1) participant characteristics, 2) components and implementation of the language interventions, and 3) indicators of study quality.

## 4 METHODS

### 4.1 Criteria for considering studies for this review

#### 4.1.1 Types of studies

This review includes randomised controlled trials (RCTs) or quasi‐experimental (QE) designs without randomisation. Control groups in the studies consisted of “business‐as‐usual” (BAU), waiting list, passive and active conditions in a domain that did not involve language and reading activities as these may have an indirect effect on other oral language skills. In addition, interventions comparing two language interventions with different delivery components or different dosage were excluded. The language intervention was required to be additional to treatment, as is usual to determine whether the focused work on language provided added value to children with neurodevelopmental disorders. In addition, we excluded single‐subject design studies as the results from these studies may not be comparable with the studies examined in our review due to the type of methodology used to assess the efficacy of language interventions. To be included, studies had to report assessments at baseline and after the completion of the training (i.e., a post‐test and/or a follow‐up) on language outcome measures. This allowed us to evaluate the following: 1) whether the intervention and control groups had comparable characteristics at the beginning of the intervention, and 2) whether the training was effective after the intervention and/or at follow‐up.

#### 4.1.2 Types of participants

Studies eligible for the review assessed samples of children from 2 to 18 years with neurodevelopmental disorders, including Developmental Language Disorder or language difficulties, autism, intellectual disability, Down syndrome, Fragile X syndrome and Williams syndrome. We also included studies in which children were described as having “language difficulties” if these difficulties were sufficiently severe on standardised assessment to warrant a diagnosis and/or the children were reported to be receiving specialist clinical or education services for language. In evaluating the eligibility of the clinical samples, we examined information on the children's clinical diagnosis or the clinical assessment and criteria for diagnosis provided by the author(s). It should be noted that there was great variability in the definition of the clinical samples, with some studies describing children's clinical diagnosis or criteria for a diagnosis and others reporting comprehensive information on the clinical assessment through cognitive, developmental and adaptive behaviour measures. In addition, there were studies of children recruited in regular schools and selected through cut‐off scores on language assessments (i.e., children with language problems) and others on children attending special schools or clinics. For this reason, we included studies that recruited from clinical caseloads or specialist education provision and/or used at least the 16th percentile (−1 SD) on receptive/expressive tests, as this is a cut‐off commonly used to identify children with language difficulties, and there is no consensus on a quantitative cut‐off for language disorder (Bishop et al., 2017). We excluded studies on children with primary speech sound disorders these are related to oral‐motor function, articulation and dyspraxia, where the primary intervention target is improving speech intelligibility (Cohen, 2001).

#### 4.1.3 Types of interventions

The included studies had to specifically target oral language skills through different techniques ranging from explicit and structured activities (i.e., explicit instruction on vocabulary, narrative structure or grammatical rules) to implicit and broad activities (i.e., shared book reading, general language stimulation). We also included interventions aimed at supporting and enhancing parents’ and teachers’ interactions with children who had neurodevelopmental disorders to optimise their language input and their responses to children's communicative attempts, to facilitate child language development. Studies were excluded if they failed to provide sufficient information on intervention content to judge the focus on oral language or where language was included as an outcome measure but the intervention itself focused on broader behavioural or developmental targets.

Since this review aimed to assess the efficacy of oral language interventions, we excluded the following:

- Interventions that were not primarily language interventions but instead targeted many areas to sustain children's joint attention, engagement, regulation and/or primarily social skills (e.g., Joint Attention, Symbolic Play, Engagement and Regulation [JASPER]).
- Interventions that targeted children's play and social skills or approaches focusing on visual and/or written information to supplement verbal communication (Treatment and Education of Autistic and related Communications Handicapped Children [TEACCH], the Picture Exchange Communication System [PECS], augmentative and alternative communication [AAC]).
- Interventions that solely targeted phonological awareness, letter knowledge, reading fluency or articulation skills.
- Interventions that focused on general cognitive skills, such as working memory, executive functions or auditory processing as intervention effects, since these tend to be limited to similar training tasks and do not transfer to specific oral language targets (Melby‐Lervåg & Hulme, 2013).
- Dietary and pharmaceutical interventions, which do not primarily target oral language skills.

#### 4.1.4 Types of outcome measures

##### Primary outcomes

The oral language outcome measures included in the review were standardised tests, observational measures, parent‐report questionnaires or researcher‐made tests assessing vocabulary, grammar, narrative, discourse processing and pragmatic language in receptive and expressive modalities. Standardised tests comprised tools measuring expressive (e.g., the Expressive Vocabulary Test—Second Edition [EVT‐2]; Williams, 2007) and receptive vocabulary (e.g., Peabody Picture Vocabulary Test—Fourth Edition [PPVT‐4]; Dunn & Dunn, 2007), grammar (e.g., Test for Reception of Grammar Version 2 [TROG‐2]; Bishop, 2003), composite scores of receptive and expressive skills derived from omnibus tests (e.g., Clinical Evaluation of Language Fundamentals—Fourth Edition [CELF‐4]; Semel et al., 2006). As for observation measures, we included the mean length of utterance (MLU). When these tests were not available, we coded parent‐report questionnaires of children's language skills (the Macarthur‐Bates Communicative Development Inventories [M‐CDI]; Fenson et al., 2007). Finally, the assessment tools for communication acts (i.e., eye contact, conversational repair, topic maintenance) were excluded because these are mixed indicators of communication and language skills.

##### Secondary outcomes

###### Duration of follow‐up

We collected data not only from immediate post‐treatment testing but also from long‐term follow‐up when available.

###### Types of settings

We included studies in which interventions were delivered in preschools, kindergartens, schools, clinical centres or the children's homes.

###### Delivery agents

We included intervention studies delivered to children with neurodevelopmental disorders by clinicians (i.e., psychologists, speech‐language therapists [SLTs] and their assistants), project staff (i.e., researchers, research assistants), parents or teaching staff (i.e., teaching assistants) that aimed to improve the oral language skills of children with neurodevelopmental disorders. Other studies were excluded from this review because they included non‐person‐delivered interventions. Specifically, computer‐assisted interventions and interventions with tablets/iPads typically include brief manipulations in experimental laboratory settings and fall outside of the traditional delivery agents targeted in this review. Animal‐assisted interventions do not target the enhancement of language, focusing instead on adaptive communication.

### 4.2 Search methods for identification of studies

#### 4.2.1 Electronic searches

The last electronic search was conducted in April 2022. In the searches, the following databases were used: MEDLINE, Embase, ERIC and PsycINFO (all cross‐searched in Ovid), CINAHL (EBSCO), the Cochrane Library, the Campbell Library, LILACS (Latin American and Caribbean Health Sciences Literature), SpeechBITE, Epistemonikos, ClinicalTrials.gov, Linguistics and Language Behavior Abstracts (LLBA), Scopus Science Direct, Web of Science and Google Scholar. The search included references without any restrictions on year or language. Retrieval experts from the medical library at the University of Oslo supervised the search. A complete list of research terms used for the present review is reported in Supporting Information 1.

#### 4.2.2 Searching other resources

We scanned the reference lists of previous reviews and meta‐analyses on language interventions for the specific diagnostic groups in the present review and conducted a hand search of the tables of contents of the following key journals: *Journal of Child Psychology and Psychiatry, Journal of Autism and Developmental Disorders, International Journal of Language and Communication Disorders*, and *Journal of Intellectual Disability Research*. Finally, we searched grey literature, including dissertations, reports and conference proceedings via OpenGrey.eu and PDF searches in Google.

### 4.3 Data collection and analysis

#### 4.3.1 Selection of studies

**Figure 2:**
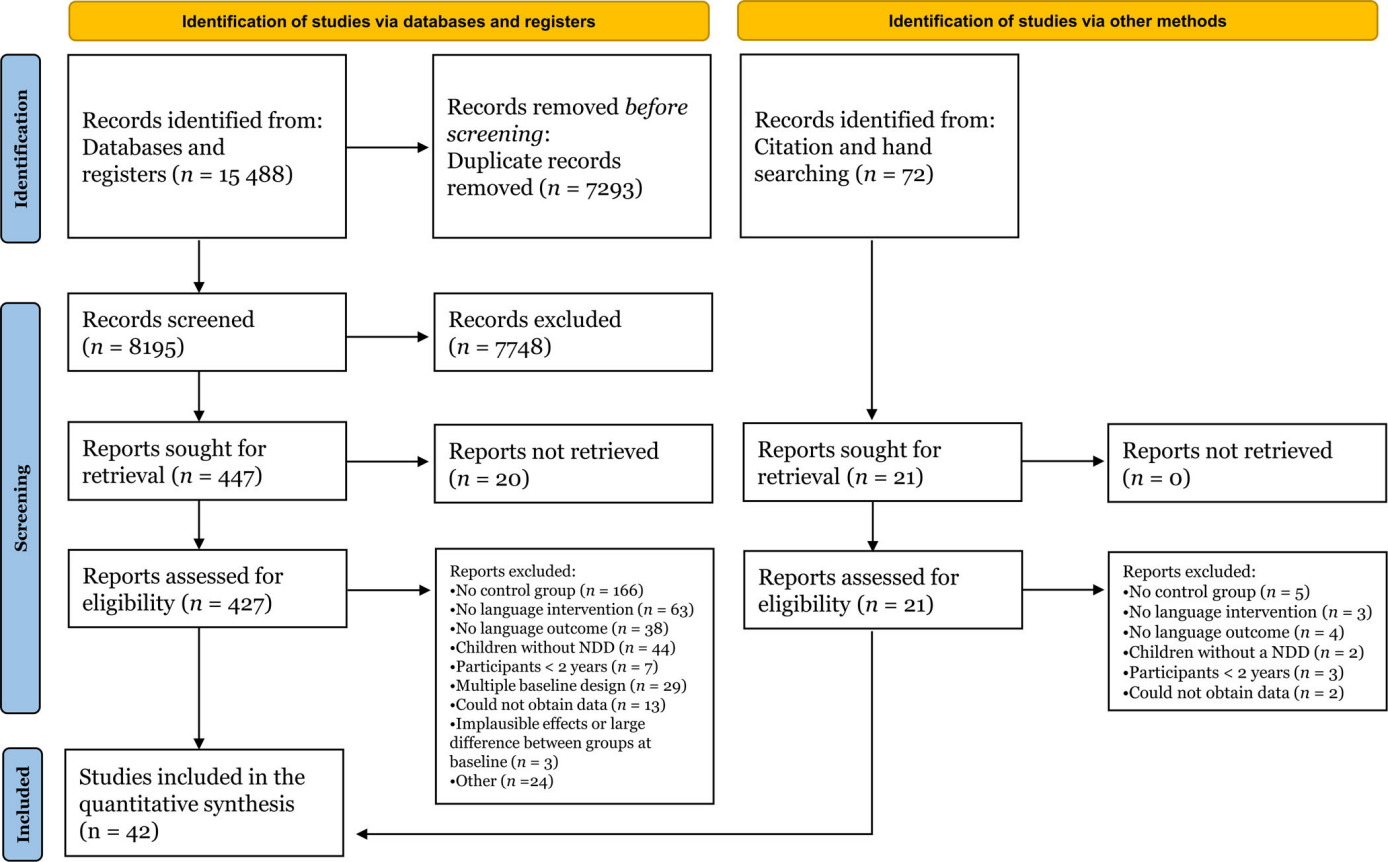
Flow diagram of the search and inclusion of references.

The meta‐analysis was pre‐registered (Nordahl‐Hansen et al., 2019) and conducted according to the Preferred Reporting Items for Systematic Reviews and Meta‐Analyses (PRISMA; Page et al., 2021). The flowchart in Figure 2 reports information regarding the overall literature search, process of study selection and final number of studies included. The search resulted in 8195 records after deduplication. The references were imported into the Rayyan software program to conduct screening by titles and abstracts. First, a random sample of 520 records was independently double‐screened by one of the authors and a trained research assistant to assess inter‐rater agreement. The inter‐rater agreement was assessed using Cohen's *K* was satisfactory, *K* = 0.82. Any conflicts and questions related to the eligibility criteria were resolved by discussion with the co‐authors before proceeding with the screening. After screening by titles and abstracts, 427 references were available; full texts were then screened to check whether the articles met our inclusion criteria. Another 21 records were identified via citation searching, resulting in a total of 448 records assessed for eligibility. At this stage, two different authors (one being the first author, the rest being distributed among the remaining authors) independently screened a random sample of 60% of the records. The inter‐rater agreement with Cohen's *K* was good (*K* = 0.83). Questions and conflicts were discussed and resolved among the authors, and doubts about the inclusion or exclusion of the remaining 40% of the papers were discussed for studies that needed further assessment and evaluation.

#### 4.3.2 Data extraction and management

The data set of effect sizes was coded in long form, with different effect sizes referring to the comparison between pre‐test and post‐test (or follow‐up) on each row. (A time lag was added to distinguish between post‐test and follow‐up scores.) Effect sizes (i.e., SMDs; see the section *Measures of treatment effects*) were calculated from descriptive statistics reported in tables or text (*N*, *M*, SD) for the intervention and control groups at the pre‐ and subsequent assessments (i.e., post‐test, follow‐up) whenever possible. Several of the studies in this review evaluated the efficacy of the intervention on more than one language outcome measure. When this was the case, all outcome measures of interest were coded. When studies compared the same control group to different treatment groups, we computed the effect size differences between each eligible treatment group and the same control group. Finally, study findings were sometimes reported in multiple reports, and authors were contacted if there was uncertainty about multiple publications of the original study. In addition, if more than one study was described in one report, the findings for each study were coded separately.

We coded information on participants’ characteristics, including the type of disorder and diagnostic status, mean age (in months), sex (proportion of boys), non‐verbal IQ and language level at baseline, when available. This information was coded separately for each group, when possible, and then pooled. As for the components and implementation of the language interventions, information on the type of intervention, the focus of the intervention and individual effect sizes included the language outcome measures and the type of indicator provided. In addition, information on settings, delivery agents, session structure of the intervention (individual vs. group intervention) and dosage—defined as session duration (in minutes), the total number of sessions and the number of weeks of intervention—was coded. As for study quality, the modality of recruitment of participants, study design, the status of the control group, type of language test, country, year and type of publication were coded. The whole sample of studies was used to calculate the inter‐coder agreement on coding. To accomplish this, the first and third author coded *N*, *M*, and SD data for the intervention and control groups for pre‐/post‐testing or follow‐up. Once each coder calculated the effect sizes based on the coded data, Pearson's correlation was used to test inter‐coder agreement. The Pearson's correlation between effect sizes was high (*r* = 0.87), indicating good agreement. Any disagreements were resolved by discussion and consultation of the original article.

#### 4.3.3 Assessment of risk of bias in included studies

Studies included in the review were evaluated using the Cochrane Collaboration's scale for assessing risk of bias in randomised trials (Higgins et al., 2011). The tool identifies five quality indicators examining *selection bias* (i.e., sequence generation and allocation concealment), *performance bias* (i.e., participants and personnel unaware of group assignment), detection bias (i.e., blind outcome assessors), *attrition bias* (i.e., participants’ withdrawals leading to incomplete outcome data) and *reporting bias* (i.e., selective outcome reporting). Each indicator was rated as high risk, unclear risk or low risk. Two of the authors independently assessed these indicators for each study, and discrepancies were discussed and solved by consensus among the authors.

#### 4.3.4 Measures of treatment effect

The effect sizes were standardised mean differences (SMDs) with Hedges’ correction for small samples (Borenstein et al., 2011). The effects were calculated as the gain observed in the intervention group (post‐test or follow‐up minus baseline) corrected for the gain in the control group, standardised by the pooled standard deviation at baseline. Specifically, effect sizes and their variances were calculated using the formulae recommended by Morris (2008; cf. index reported as *d*_ppc2_). For the calculation of the effect size variance, we adjusted the values for a pre‐/post‐test correlation of *ρ* = 0.5 as a reasonable estimation of pre‐post correlations in training studies. Effect sizes calculated from variables with a negative scoring (i.e., with higher values indicating worse language ability, such as error counts) had their sign inverted. Thus, a positive effect size indicated that the intervention group receiving oral language skills training showed a larger pre‐/post‐test gain compared with the control group. As for the size of the effect, this was evaluated against baseline benchmarks for effect sizes from studies of preK–12 education interventions evaluating effects on student achievement by Kraft (2020). Based on the distribution of 1942 effect sizes from 747 RCTs evaluating education interventions with standardised test outcomes, Kraft (2020) suggested the following benchmarks: less than 0.05 is *small*, 0.05 to less than 0.20 is *medium* and 0.20 or greater is *large*.

#### 4.3.5 Unit of analysis issues

Multiple outcome variables of interest were frequently reported for the same intervention–control group comparison, and multiple comparisons were sometimes reported in the same study (i.e., because a study included two different intervention groups compared against one control group; see the section on *Data Synthesis*). Therefore, effect sizes were coded as nested within a group comparison and the latter as nested within a study. This structure of dependencies was dealt with in the data analysis using correlated and hierarchical models, with an assumed constant correlation of *ρ* = 0.7 among effect sizes clustered within the same study (see the section on *Data Synthesis*).

#### 4.3.6 Dealing with missing data

When the descriptive statistics (*N, M, SD*) necessary for calculating the effect sizes for any of the outcome measures of interest were not available from the text or tables, data were extracted from the figures and elaborated on if necessary (i.e., SDs were approximated from plotted error bars representing standard errors or confidence intervals and *N*, if not directly reported). If needed information was still missing, the authors were contacted. When eligible references presented a potential overlap (i.e., they were published by the same research group and described similar language interventions and participants), the authors were contacted to clarify whether the records could describe the same study. When it was clear that different references reported the same data, only records with more information (i.e., outcome measures or data on moderator variables) were included.

#### 4.3.7 Assessment of heterogeneity

Variability in effect sizes and heterogeneity between studies were quantified using different statistics (Borenstein et al., 2017) applied to the meta‐analytic models (see details in the section on statistical modelling). First, we used the *Q*‐statistic to test the null hypothesis that there is no variability in the underlying true effect size (either between or within studies). Second, we reported the *I* ^2^ index to indicate how much of the observed variance was estimated to reflect differences in the true effect sizes rather than sampling error. Third, we provided the estimated standard deviation of the true effect sizes between studies, *τ*_study_, between comparisons, *τ*_comparison_, and between effects, *ω*.

#### 4.3.8 Assessment of reporting biases

Assessment of publication bias was complicated by the complex structure of the data and by the predictably large heterogeneity. A complex, multilevel data structure implies that a publication bias may arise for different reasons, including one or more single non‐significant outcomes being omitted (or reported but *p*‐hacked) by a study, a group comparison not being reported by a study, or even an entire study not being published. We have no means of investigating all these possibilities. In addition, we did not consider the variety of inferential processes that may affect publication bias within each individual study (i.e., type of analysis, use of covariates, corrected vs. uncorrected multiple comparisons).

We employed the *p*‐curve method on the whole set of effect sizes (Simonsohn et al., 2014), as stated in the protocol. The *p*‐curve represents the plotted distribution of *p*‐values, and it depends on both the distribution of effect sizes and their sample sizes. A right‐skewed *p*‐curve (i.e., with a prevalence of small *p*‐values) suggests a true non‐zero effect size, whereas a *p*‐curve that is left‐skewed with a prevalence of *p*‐values just below *p* = 0.05 suggests publication bias. Unfortunately, the *p*‐curve method may present the problem of not accounting for the presence of dependency structures between the effect sizes, and it is known to perform poorly when there is between‐study heterogeneity (Rodgers & Pustejovsky, 2021). The former (but not the latter) problem can be tackled using methods based on meta‐regression. Among them, we chose the precision‐effect test and precision‐effect estimate with standard errors (PET‐PEESE) method, which is known to perform comparatively better than alternative conventional meta‐analytic methods to assess publication bias (Stanley, 2017).

The PET‐PEESE consists of a two‐step meta‐regression method in which the standard error (first step) and then the variance of the effect size (second step) is used as the moderator for the effect size (Stanley & Doucouloagos, 2014). The second step is performed for a better estimate only if the first step suggests a non‐zero true effect size. The final, bias‐free estimated effect size is the intercept of the model. We implemented the PET‐PEESE method as an additional moderator analysis on the main meta‐analytic model.

A related problem with meta‐regression tests of publication bias (or the funnel plot) is that the effect size estimate in SMDs is not independent from its variance (Morris, 2008), which inflates the risk of falsely detecting or overestimating publication bias (Zwetsloot et al., 2017). Therefore, we performed the PET‐PEESE method on an alternate data set in which the variances were estimated, setting *d* = 0 in Morris’ (2008) formula. This underestimates variances for non‐zero effects, but at the same time, it presents the benefit of providing variance estimates that are orthogonal to the effect sizes and thus usable in funnel plots or regression tests.

#### 4.3.9 Data synthesis

##### Meta‐analytic modelling

R software, version 4.3.1 (R Core Team, 2023) was used to calculate effect sizes and perform all analyses except the *p*‐curve. The following R packages were used: “metafor” (Viechtbauer, 2010) to fit maximum likelihood models, “clubSandwich” (Pustejovsky, 2017) to impute covariance matrices and calculate robust standard error estimates and “ggplot” (Wickham, 2016) for plotting. Before performing the analysis, we checked for extreme values to remove any implausible effect sizes (i.e., SMD > 2 in terms of net pre‐/post‐test gain) and any very large between‐group difference at the baseline (i.e., SMD > 1 at pre‐test).

Following the guidelines by Borenstein et al. (2011), meta‐analytic estimates were obtained using random‐effects models, which allowed us to better account for the predictable heterogeneity across effect sizes. Our effects were nested within group comparisons, which were nested within the studies. Within the same study, multiple effects had correlated sampling errors. To account for this complex structure of dependencies between effect sizes, we adopted a multilevel modelling framework with correlated and hierarchical effects (CHE models; Pustejovsky & Tipton, 2021). A three‐level random‐effects structure was set with random intercepts for studies, group comparisons and individual effects. The structure of variances was passed to the model via an imputed block‐diagonal covariance matrix (Pustejovsky & Tipton, 2021), which assumed a constant correlation of *ρ* = 0.7 among the effect sizes clustered within the same study. Alternative values of *ρ* had negligible effects on the final meta‐analytic estimates (except for the heterogeneity being attributed to variability in the effects between vs. within studies). Coefficients were also estimated via maximum likelihood.

##### Additional analysis of power

Based on the results of the meta‐analysis and plausible assumptions, we retrospectively considered the power of the set of studies included in our review. This served both to clarify whether the extant literature had enough statistical power for the threshold of evidence traditionally used in the psychological literature (with critical *α* = 0.05 for type I error) and to provide guidelines for future studies. Considering power is important not only to reduce false negative results and improve the discriminability between true positive and false positive results but also to reduce the mean overestimation of truly non‐zero effect sizes that emerge as statistically significant (i.e., Altoè et al., 2020; Gelman & Carlin, 2014).

In controlled trials with pre‐/post‐test comparisons, power depends not only on sample size (i.e., how many participants are allocated to each group) but also on the reliability of the measures in terms of their stability over time. Toffalini et al. (2021) recently showed that if used appropriately, such reliability could be largely improved with even a few repeated measurements per time point. The retrospective power analysis was performed via simulation using the analytic strategy and code provided by Toffalini et al. (2021). We assumed good (but not excellent) stability of measures (test/re‐test correlation of *ρ* = 0.7), and we set a critical *α* = 0.05 for significance, assuming a single comparison. (With multiple testing, correction should be applied to *p*‐values, but we did not investigate this case.) We examined the power reached with various combinations of effect sizes (as net SMDs) and sample sizes. All simulations were performed with 5000 iterations.

#### 4.3.10 Subgroup analysis and investigation of heterogeneity

Moderator analysis was conducted via meta‐regression. Since studies do not always report values for all moderators of interest, there is a predictable loss of information; because of this, we chose to limit the moderator analysis only to moderators for which there was a subset of at least *k* = 5 studies with complete information. For categorical moderator variables, we performed moderator analysis only on levels of the moderator that were represented by at least *k* = 5 studies. It should be noted that the moderator analysis was conducted only for gains observed in the post‐test because of the limited number of studies that also presented follow‐up observations.

The moderators examined in the meta‐analysis were as follows:

*Participants’ characteristics*

- *Type of disorder*. Information on the type of disorder was coded as language disorder, autism, intellectual disability, Down syndrome, Fragile X syndrome or Williams syndrome according to the clinical diagnosis or the clinical assessment and criteria for a diagnosis provided by the author(s).
- *Diagnostic status*. We examined whether the children reported a “clinical diagnosis” or “difficulties.” When children with autism were identified with diagnostic tools or evaluated according to autism symptomatology and when participants were described as having a genetic syndrome (i.e., Down syndrome, Fragile X syndrome, Williams syndrome), we coded this information as “clinical diagnosis.” Since there was considerable variation in the definition of language disorder, children performing below the 10th percentile on standardised tests evaluating language skills, children in special schools for children with language disorders or children referred from speech‐language therapy caseloads were coded as “clinical diagnosis,” whereas those selected by screening from mainstream classrooms performing below the 16th percentile were given the label “difficulties.”
- *Age*. The mean age (in months) of the intervention and control groups was coded.
- *Sex*. Sex composition was coded by calculating the proportion of boys in the overall sample (children in the intervention[s] and control groups).
- *IQ*. Scores on standardised IQ test batteries assessing non‐verbal IQ were evaluated. As measures of non‐verbal IQ, we found several tests, including the Leiter International Performance Scale‐Revised (Leiter‐R, 1979; Roid & Miller, 1997), the performance score of the Wechsler Intelligence Scale for Children—Fourth Edition (WISC‐IV; Wechsler, 2003), the Stanford‐Binet Intelligence Scales—Fifth Edition (SB‐5; Roid, 2003), and the Test of Nonverbal Intelligence—Third Edition (Brown et al., 1997), and Kaufman Brief Intelligence Test‐2 (KBIT‐2; Kaufman & Kaufman, 2004). We coded all scores as standardised scores with the IQ metrics (i.e., with *M* = 100 and SD = 15 for the normative population) to obtain data on a comparable scale.
- *Language level at baseline*. Scores on standardised language test batteries that served to evaluate children's overall or receptive language skills at baseline were coded, such as the British Picture Vocabulary Scale—Second Edition (BPVS‐II; Dunn et al., 1997), the Comprehensive Assessment of Spoken Language (CASL; Carrow‐Woolfolk, 1999) and the Bayley language composite (Bayley, 2006). Thus, we coded the standardised scores transformed into the metrics of IQ scores.

*Components and implementation of language interventions*

- *Type of intervention*. Interventions were coded as “explicit instruction,” “implicit/incidental programmes” and “hybrid.”
- *Focus of the intervention*. The focus of the language intervention was coded as targeting vocabulary, grammar or multi‐component programmes when directed at more than one language skill. In addition, language interventions could focus on general language stimulation and book reading–related, narrative or social communication skills (i.e., pragmatic language).
- *Language outcome measure*. The language task considered to evaluate the efficacy of the intervention was coded in “vocabulary (expressive or receptive), grammar, discourse (expressive or receptive language sub‐scales), and omnibus tests.
- *Settings*. The place where the intervention was delivered was coded as “clinic,” “school” (school or special schools/classrooms), “preschool” (nursery, kindergarten) or “home.”
- *Delivery agents*. When the intervention targeted the child directly, those who led the intervention were categorised as “clinicians” (SLTs or psychologists) or “project staff” (researchers or trained research assistants). For interventions in which parents and teachers were trained to deliver the intervention to the children, the delivery agent was coded as “parent‐mediated” or “teacher‐mediated” (teachers or teaching assistants).
- *Session structure of the intervention*. Interventions delivered one‐on‐one with the child or parents were coded as “individual intervention,” whereas those implemented in groups were classified as “group intervention.”
- *Dosage*. The session duration (in minutes), the total number of sessions and the number of weeks of intervention were coded. However, for many implicit/incidental and hybrid interventions (especially those parent‐mediated interventions provided for children with autism), total intervention hours do not reflect the total amount of time spent focusing on language, which was not possible to determine.

*Indicators of study quality*

- *Recruitment*. Information on the modality of recruitment of participants was coded into “specialised centres,” including children on specialist waiting lists and those involved in training programs/therapy caseloads, “schools,” “special schools” and “local agencies and advertisement.”
- *Study design*. Information on the randomisation was coded as “RCT” and “QE.”
- *Status of the control group*. The control group was defined as “active,” “waiting list” (delayed treatment) or “BAU.”
- *Type of language test*. This moderator was coded as “standardised test,” “observational measure,” “parent‐report questionnaire” or “researcher‐made test.”
- *Country*. The country where the study took place was coded as “Europe,” the “US” or “other” (Canada, China, India, Pakistan, Malaysia and Australia).
- *Year of publication*. The publication year of all records was coded.
- *Type of publication*. Studies were coded as “published” (papers in peer‐reviewed journals) or “unpublished” (theses and conference papers).

#### 4.3.11 Sensitivity analysis

For the main meta‐analytic estimates, a sensitivity analysis was conducted to see how much the estimate varied with each individual study. This was obtained by recalculating the estimate and removing one single study at each iteration. A second sensitivity analysis was conducted to determine how much excluding studies with implausibly large effects has affected the main meta‐analytic estimates.

##### Treatment of qualitative research

We did not include qualitative research.

#### 4.3.12 Summary of findings and assessment of the certainty of the evidence

See the discussion section for a detailed account of this.

## 5 RESULTS

### 5.1 Description of studies

#### 5.1.1 Results of the search

After completion of the full‐text screening and preliminary check, we retained 42 publications for the analysis (see section below). A detailed description of the effect sizes and group comparisons derived from these publications are reported in the sections below.

#### 5.1.2 Included studies

##### Overview of study characteristics

A total of 141 effects from 45 studies and 54 group comparisons were coded. There were only six studies with multiple group comparisons, with 39 including only one pair of groups (one intervention vs. one control). A preliminary check led to 12 effects (8.5%) being removed as implausible (these ranged between 2.1 and 5.70, with a single outlier of 15.7 being due to a floor effect with virtually null between‐participant variability at pre‐test). An additional three effects (2.4%) were removed because of a large difference between groups at baseline (absolute values ranged between 1.1 and 2.2). Thus, three studies (Allen & Marshall, 2015; Girolametto et al., 1996; Lousada et al., 2016) were excluded from the analysis at this stage. After this filtering, the final data set included 129 effect sizes from 42 studies and 51 group comparisons. (Again, only six studies included more than one group comparison.) An overview of the characteristics of the studies included in the review is reported in Table 1.

**Table 1:** Characteristics of included studies.

Study	NDD, diagnostic status	Age	Boys (%)	Type of intervention	Intervention focus	Settings	Delivery agents	Session structure	Dosage	*N*	CG status
Adams et al. (2012)	Pragmatic language disorder +ASD, Clinical	6–11 years	86.21	Explicit instruction	Social communication skills	School	Clinicians	Individual	16–20 sessions × 60 min each over 13 weeks	TG = 59 vs. CG = 28	BAU
Baxter et al. (2022)	DS, Clinical	7–11 years	N/A	Hybrid	Grammar: Morphosyntax ‐ past‐tense ‐ed	School	Teacher‐mediated	Individual	42.5 sessions × 20 min each over 10 weeks	TG = 26 vs. CG = 26	Waiting list
Boyle et al. (2009)	LD, Clinical	6–11 years	77.00	Hybrid	Multi‐component: Comprehension monitoring, vocabulary, grammar and narrative	School	Clinicians	Group vs. individual	45 sessions × 30–40 min each over 15 weeks	Direct individual: *N* = 34 Direct group: *N* = 31 Indirect individual: *N* = 33 Indirect group: *N* = 32 vs. CG = 31	BAU
Burgoyne et al. (2012)	DS, Clinical	5–10 years	49.12	Explicit instruction	Vocabulary	School	Teacher‐mediated	Individual	100 sessions × 40 min each over 20 weeks	TG = 28 vs. CG = 26	Waiting list
Calder et al. (2021)	LD, Clinical	5–6 years	72.00	Explicit instruction	Grammar: Morphosyntax ‐ past‐tense ‐ed	School	Clinicians	Individual	10 sessions × 20–30 min each in 10 weeks	TG = 10 vs. CG = 11	Waiting list
Casenhiser et al. (2013)	Autism, Clinical	2–4 years	N/A	Implicit/incidental	Social communication skills	N/A	Parent‐mediated	Individual	48 sessions × 120 min each over 48 weeks + parent‐training sessions every 8 weeks	TG = 25 vs. CG = 26	BAU
Crain‐Thoreson & Dale (1999)	LD, Clinical	3–5 years	68.75	Implicit/incidental	Vocabulary	Home vs. school	Parent‐mediated vs. teacher‐mediated	Individual	32 sessions × 90 min each over 8 weeks	Parent‐mediated: *N* = 10 Teacher‐mediated: *N* = 13 vs. CG = 9	Active
Ebbels et al. (2012)	LD, Clinical	9–15 years	73.00	Explicit instruction	Vocabulary	School	Clinicians	Individual	8 sessions × 30 min each over 8 weeks	TG = 8 vs. CG = 6	Waiting list
Ebbels et al. (2014)	LD, Clinical	11–15 years	71.43	Explicit instruction	Grammar: Morphosyntax ‐ past‐tense ‐ed	Clinic	Clinicians	Individual	8 sessions × 15 min each over 8 weeks	TG = 6 vs. CG = 7	Waiting list
Fey et al. (1993)	LD, Clinical	3–5 years	74.50	Parent: hybrid; Clinician: explicit	Grammar	Home vs. clinic	Parent‐mediated vs. Clinicians	Individual+group	Parent‐mediated: 12 sessions × 120 min each over 20 weeks; Clinicians: 60 min each over 20 weeks	Parent‐mediated: *N* = 10 Clinicians: *N* = 11 vs. CG = 9	Waiting list
Gallagher & Chiat (2009)	LD, Clinical	3–5 years	75.00	Hybrid	Multi‐component: Vocabulary and grammar	Clinic vs. preschools	Clinicians	Group	Intensive condition: 24 sessions × 240 min each over 24 weeks; Nursery‐based: 12 sessions × 60 min each over 12 weeks	Intensive: *N* = 8, Nursery‐based: *N* = 8 vs. CG = 8	Waiting list
Gengoux et al. (2019)	Autism, Clinical	2–5 years	88.37	Explicit instruction	General language stimulation	Home	Parent‐mediated	Individual	Intensive phase: 12 sessions × 60 min each + 12 children in‐home sessions in 12 weeks; Maintenance phase: monthly parent training and weekly in‐home sessions for 20 weeks	TG = 23 vs. CG = 20	Waiting list
Green et al. (2010)	Autism, Clinical	2–5 years	90.79	Implicit/incidental	General language stimulation	Clinic	Parent‐mediated	Individual	18 sessions × 120 min over 24 weeks + booster: 6 sessions over 24 weeks	TG = 74 vs. CG = 72	BAU
Haley et al. (2017)	LD, Difficulties	3 years	53.50	Explicit instruction	Multi‐component: Vocabulary, listening comprehension, narrative	School	Teacher‐mediated	Group	42 sessions × 20 min each over 15 weeks	TG = 52 vs. CG = 51	Waiting list
Hardan et al. (2015)	Autism, Clinical	2–6 years	75.00	Explicit instruction	General language stimulation	Clinic	Parent‐mediated	Individual	12 sessions over 12 weeks	TG = 25 vs. CG = 22	BAU
Henry & Solari (2020)	Autism, Clinical	6 years	81.40	Explicit instruction	Multi‐component: Vocabulary, language comprehension	School	Teacher‐mediated	Group	72 sessions × 30 min each over 16 weeks	TG = 22 vs. CG = 21	BAU
Hudson et al. (2017)	Autism, Clinical	3–5 years	81.00	Explicit instruction	Book reading–related programme	School	Mix: Teachers + psychologists	Individual	60.12 sessions × 7–15 min each over 8 weeks	TG = 47 vs. CG = 44	BAU
Joffe et al. (2019)	LD, Difficulties	12 years	63.13	Explicit instruction	Vocabulary vs. narrative vs. multi‐component	School	Teacher‐mediated	Group	18 sessions × 45‐60 min each over 6 weeks	Vocabulary: *N* = 82 Narrative: *N* = 84 Combined: *N* = 84 vs. CG = 83	Waiting list
Lavelli et al. (2019)	LD, Clinical	3–5 years	71.88	Hybrid	Book reading–related programme	Home	Parent‐mediated	Individual + group	32 sessions over 9 weeks + parent‐training: 3 small‐group sessions of 180 min each and 3 individual sessions of 60 min each with video feedback	TG = 20 vs. CG = 12	Waiting list
Lo & Shum (2021)	Autism, Clinical	3–6 years	83.87	Hybrid	Book reading–related programme	Home	Parent‐mediated	Group	12 sessions × 10–15 min each over 6 weeks + parent‐training workshop	TG = 17 vs. CG = 14	Active
Lourenço et al. (2020)	LD, Clinical	5 years	N/A	Explicit instruction	Narrative skills	Clinic	Clinicians	Individual	8 sessions × 60 min each over 8 weeks	TG = 7 vs. CG = 7	BAU
McDuffie et al. (2018)	FXS, Clinical	10–16 years	100.00	Implicit/incidental	Book reading–related programme	Home	Parent‐mediated	Individual	12 sessions over 12 weeks	TG = 10 vs. CG = 10	BAU
Motsch & Marks (2015)	LD, Clinical	9 years	68.79	Explicit instruction	Multi‐component: Vocabulary and/or syntax	School	Clinicians	Individual vs. group	Individual condition: 20 sessions × 30 min each over 20 weeks; Group condition: 20 sessions × 45 min each over 20 weeks	Individual: *N* = 40 Small groups: *N* = 38 vs. CG = 45	BAU
Motsch & Ulrich (2012)	LD, Difficulties	3–4 years	58.82	Explicit instruction	Vocabulary	Preschools	Clinicians	Individual	13 sessions × 30 min each over 5 weeks	TG = 26 vs. CG = 25	BAU
Pile et al. (2010)	LD, Clinical	3–5 years	61.11	Explicit instruction	Book reading–related programme	Home	Clinicians	Group	1 introductory parent session + 8 group sessions × 60 min each (+15 min parents’ follow‐up) over 9 weeks	TG = 19 vs. CG = 17	Waiting list
Rahman et al. (2016)	Autism, Clinical	2–9 years	81.54	Implicit/incidental	General language stimulation	Clinic	Parent‐mediated	Individual	12 sessions × 60 min over 12 weeks	TG = 29 vs. CG = 30	BAU
Roberts & Kaiser (2012)	LD, Clinical	2–3 years	79.00	Implicit/incidental	General language stimulation	Home	Parent‐mediated	Individual	24 sessions × 60 min each over 12 weeks + 4 parent‐training workshops	TG = 16 vs. CG = 18	BAU
Roux et al. (2015)	Autism, Clinical	6–12 years	82.22	Explicit instruction	Multi‐component: Vocabulary, language comprehension	School	Research staff	Group	48 sessions × 30 min each over 16 weeks	TG = 24 vs. CG = 21	BAU
Sajaniemi et al. (2010)	LD, Clinical	3–5 years	76.19	Implicit/incidental	Multi‐component	Clinic	Clinicians	Group	44 sessions (2× week) but length of session not reported	TG = 22 vs. CG = 20	BAU
Salt et al. (2002)	Autism, Clinical	3 years	82.35	Implicit/incidental	General language stimulation	Clinic	Parent‐mediated	N/A	Unclear	TG = 12 vs. CG = 5	Waiting list
Sepúlveda et al. (2013)	DS, Clinical	6–14 years	55.00	Explicit instruction	Grammar	Clinic	Clinicians	Individual	30 sessions × 30 min each over 14 weeks	TG = 10 vs. CG = 10	BAU
Sokmum et al. (2017)	Autism, Clinical	4 years	87.10	Implicit/incidental	General language stimulation	Clinic	Parent‐mediated	Individual	11 sessions (8 group training + 3 visit home) over 12 weeks	TG = 16 vs. CG = 15	BAU
Solari et al. (2020)	Autism, Clinical	5–11 years	91.67	Explicit instruction	Book reading–related programme	School	Research staff	Group	70 sessions × 30 min each over 17–18 weeks	TG = 6 vs. CG = 6	BAU
Solomon et al. (2014)	Autism, Clinical	2–5 years	82.05	Implicit/incidental	General language stimulation	Home	Parent‐mediated	Individual	12 sessions × 180 min over 52 weeks	TG = 64 vs. CG = 64	BAU
Starling et al. (2012)	LD, Difficulties	12–14 years	77.00	Hybrid	General language stimulation	School	Teacher‐mediated	Group	10 sessions × 50 min each over 10 weeks	TG = 21 vs. CG = 22	Waiting list
van der Schuit et al. (2011)	ID + DS, Clinical	2–6 years	60.71	Implicit/incidental	General language stimulation	Clinic+Home	Teachers‐mediated	Group	45 sessions × 150–180 min over 9‐week anchored cycle ‐ intervention lasted 2 years	TG = 10 vs. CG = 18	BAU
van Kleeck et al. (2006)	LD, Clinical	3–5 years	56.67	Hybrid	Book reading related program	Clinic	Research staff	Individual	16 sessions × 15 min each over 8 weeks	TG = 15 vs. CG = 15	BAU
Wake et al. (2013, 2015)	LD, Difficulties	4–6 years	66.02	Explicit Instruction	Multi‐component: phonological awareness, expressive language, shared book reading	Home	Parent‐mediated	Individual	Three 6‐week blocks of 18 sessions starting every 3 months	TG = 101 vs. CG = 99	BAU
Washington et al. (2011)	LD, Clinical	3–4 years	20.59	Explicit instruction	Grammar	Clinic	Clinicians	Individual	10 sessions × 20 min each over 10 weeks	TG = 11 vs. CG = 12	Waiting list
Westerveld et al. (2021)	Autism, Clinical	3–5 years	81.25	Implicit/incidental	Book reading–related programme	Home	Parent‐mediated	Individual	4 coaching sessions (+4 phone calls) ×45 min over 8 weeks	TG = 9 vs. CG = 7	Waiting list
Wright et al. (1993)	LD, Clinical	8–14 years	83.33	Explicit instruction	Vocabulary	School	Clinicians	N/A	15 sessions × 30 min each over 5 weeks	TG = 8 vs. CG = 8	N/A

Abbreviations: BAU, business‐as‐usual; CG, control group; DS, Down syndrome; FXS, Fragile X syndrome; LD, language disorder; N, sample size; N/A, not available; NDD, neurodevelopmental disorders; TG, Treatment group.

###### Participants’ characteristics

Out of 42 studies, 22 reported samples with language disorders or language problems, 14 described children with autism, three described children with Down syndrome, one described children with Fragile X syndrome, and two reported on mixed cases. Among studies with mixed cases, one intervention focused on children with intellectual disability and Down syndrome, and one included individuals with autism, pragmatic language impairment or social communication disorder, but none targeted children with Williams syndrome. As for clinical status, 36 studies included participants with a clinical diagnosis, whereas six focused on children with language difficulties who had not yet received a formal diagnosis (i.e., children with language difficulties). The unweighted grand average of mean ages across the 42 studies was 6.5 years, ranging between 2.6 and 14.2 years; the median percentage of males in the samples was 75.0%, ranging between 20.6% and 100.0%. The estimated mean non‐verbal IQ (standardised score) of participants was reported by 14 studies, with an unweighted grand average IQ = 84.68, ranging between 41.5 and 107.7. The average global language levels (standardised score) of participants at baseline were reported by 10 studies, with an unweighted grand average standard score of 80.93, ranging between 70.76 and 91.95.

###### Components and implementation of language interventions

As for the type of language intervention, 23 studies implemented explicit instruction, 12 studies used an implicit/incidental approach and eight used a hybrid approach. Some interventions focused on specific language skills (seven studies on vocabulary and six on grammar), and two studies were multi‐component programmes targeting more than one language skill at the same time. In addition, 10 studies implemented a general language stimulation intervention and eight studies implemented a book reading–related language programme. Finally, two studies focused on narrative skills and two on social communication skills. Studies examined in this review often included multiple language outcome measures, including vocabulary (16 studies on expressive vocabulary and six studies on receptive vocabulary), grammar (16 studies), discourse (15 studies on expressive discourse and 121 studies on receptive discourse), or omnibus measures (seven studies on total receptive language and seven studies on total expressive language).

Regarding the implementation of the language interventions, training was conducted at schools (16 studies), the clinic (13 studies) or home (12 studies). However, one study failed to report any information on settings (Casenhiser et al., 2013). Implicit/incidental interventions were sometimes conducted in the clinic with some sessions delivered at home. Interventions were led by clinicians (15 studies) and research staff (three studies) who worked directly with the children. In addition, there were parent‐mediated (17 studies) and teacher‐mediated (eight studies; one study was both parent‐ and teacher‐mediated) interventions delivered individually (27 studies), in groups (13 studies) or by mixed delivery (two studies). Information on dosage shows that interventions aimed directly at children and teacher‐mediated interventions lasted, on average, 41.41 min per session (ranging between 15 and 165 min) and that a mean of 29.47 sessions (ranging between 8 and 100) were delivered for an average of 12.81 weeks (ranging from 5 to 22).

###### Indicators of study quality

We examined several indicators of study quality. Participant recruitment was conducted through schools (six studies) and special schools (11 studies), specialised centres (14 studies), local agencies and advertisements (six studies) or mixed modalities (three studies), while two failed to report information (Salt et al., 2002; van der Schuit et al., 2011). Studies described RCT interventions (21 studies) and QE (21 studies) study designs involving active (two studies), waiting list (16 studies) and BAU (23 studies) groups. However, one study failed to report clear information on the control group's condition (Wright et al., 1993). As for the type of language test to evaluate the efficacy of the intervention, studies used standardised tests (32 studies), observation measures (seven studies), parent‐report questionnaires (six studies) or research‐made tasks (one study) (some used multiple types). Studies were conducted in Europe (19 studies), the US (11 studies) or other countries (12 studies), and all studies were published in peer‐reviewed journals.

#### 5.1.3 Excluded studies

We excluded studies that did not present an intervention targeting language skills, did not report any measures of language skills, did not target children with neurodevelopmental disorders or did not include an adequate control group. In addition, language intervention studies on children younger than 2 years and those using multiple baseline designs were excluded. Some studies were shortlisted but ultimately excluded because they failed to report relevant statistics to calculate an effect size, and it was not possible to obtain data from the authors (Figure 2).

### 5.2 Risk of bias in included studies

**Figure 3:**
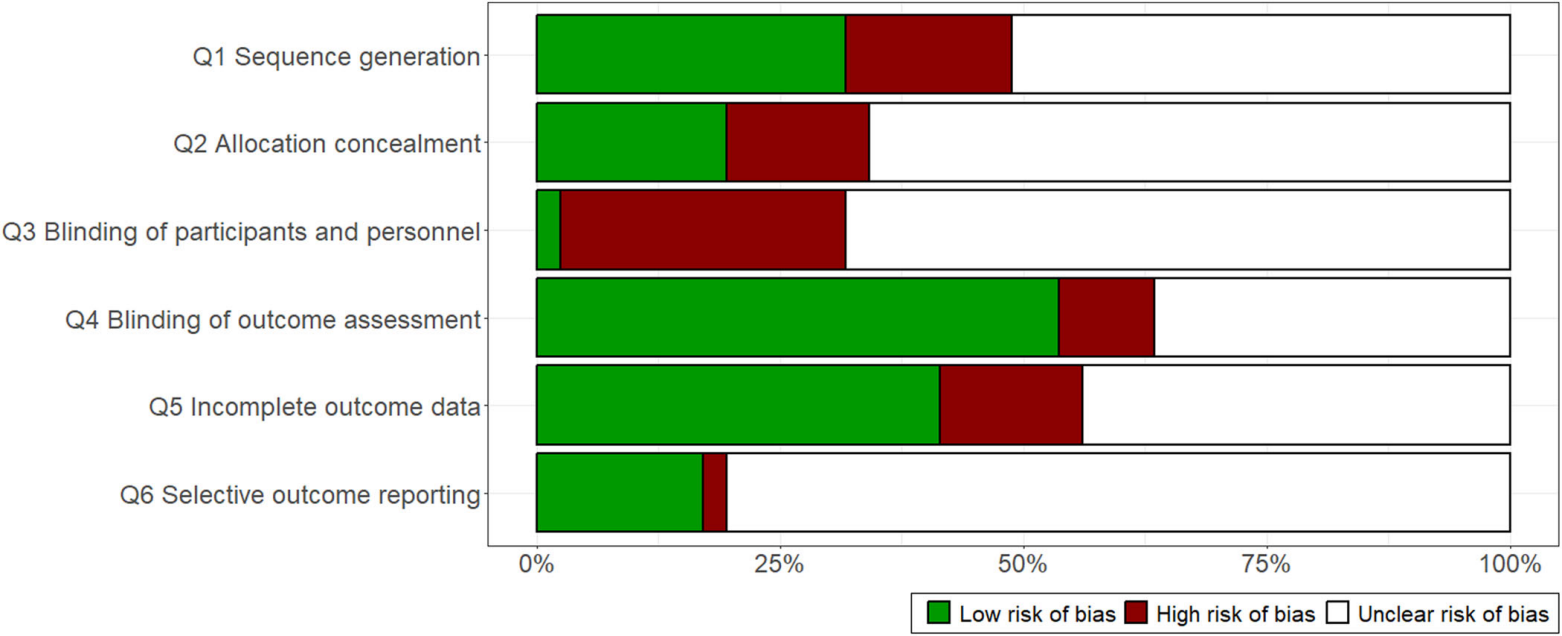
Authors’ evaluations regarding each risk‐of‐bias item presented as percentages across included studies.

Figure 3 shows an overview of the risk of bias in the included studies. Online Supplement 2 provides details on the judgements and classification of the risk of bias categories.

#### 5.2.1 Allocation (selection bias)

##### Selection bias

Sequence generation refers to random group assignment and allocation concealment and whether the participants were randomly assigned to the study conditions. When a study reported that it was impossible to randomise participants or that the study was conducted on a convenience sample, both sequence generation and allocation concealment were rated as “high risk of bias.” Although several studies labelled their intervention as randomised, many failed to report information about sequence generation and allocation concealment. In addition, some studies described a random sequence to assign participants to different conditions in studies on small sample sizes (i.e., 10 or fewer children per group). In these cases, the studies were rated as “unclear risk of bias.” When clear information on sequence generation and allocation concealment was provided, the study was rated as “low risk of bias.” Regarding sequence generation, most of the studies included in the review were rated as unclear‐risk (21 studies), whereas the others were rated as low (13 studies) or high risk (seven studies). As for allocation concealment, most of the studies were rated as “unclear risk” (27 studies) and a few as low (eight studies) or high risk (six studies).

#### 5.2.2 Blinding (performance bias and detection bias)

##### Performance bias

Studies were rated as having “high risk of bias” when the study participants and personnel conducting the study were not masked to treatment allocation. Studies that failed to report any information on blinded personnel or participants after enrolment in the study were rated as “unclear risk of bias.” Most of the included studies were rated as “unclear risk” (28 studies) and others as high (12 studies) or low (one study) risk.

##### Detection bias

Studies were rated as “high risk of bias” when the outcome evaluators were not blind to the participant's group condition and as “unclear risk of bias” when there was no information on whether the assessors were blind to the participants’ intervention group. When a study stated that outcome assessors were blinded to the participant's group assignment, the study was rated as “low risk of bias.” Approximately half the studies were rated as “low risk” (22 studies), whereas the others were rated as “unclear” or “high risk” (15 and four studies, respectively).

#### 5.2.3 Incomplete outcome data (attrition bias)

##### Attrition bias

Attrition bias refers to participants’ withdrawals, leading to missing data. When studies reported a high attrition rate and did not account for missing data in the analysis (i.e., they used multiple imputations or estimators for missing data), the study was rated as “high risk of bias.” When there was no information on the attrition rate or how missing data were treated, the study was rated as “unclear risk of bias.” In contrast, when information was available on how missing data were handled in the analysis and when the attrition rate was low, the study was rated as “low risk of bias.” Several studies were rated as low (17 studies) or unclear risk (18 studies), and a few as “high risk” (six studies).

#### 5.2.4 Selective reporting (reporting bias)

##### Reporting bias

When the intervention study was pre‐registered, the study was coded as “low risk of bias”; and when this information was not reported, the study was rated as “unclear risk of bias.” Most of the studies were rated as “unclear risk” (33 studies) and the others as “low risk” (seven studies) or “high risk” (one study, which clearly stated that the intervention was not pre‐registered).

### 5.3 Effects of interventions

#### 5.3.1 Meta‐analytic estimates

Effect sizes for pre‐test/post‐test comparisons were reported by 38 studies, with 46 group comparisons and a total of 108 effects. The overall meta‐analytic estimate of the mean effect was *d* = 0.27 [95% confidence interval [CI]: 0.15, 0.38]. In line with benchmarks suggested by Kraft (2020), this can be considered a moderate to large effect. The overall true heterogeneity was moderate, *I* ^2^ = 45%. The model does not estimate heterogeneity in the true effect size across studies, *τ*_study_ = 0.00 (meaning that the 95% PI of the true effect size across studies is estimated as having a null range), while the heterogeneity was considerable across group comparisons, *τ*_comparison_ = 0.13, and between effect sizes within the same group comparison, *ω* = 0.21.

**Figure 4:**
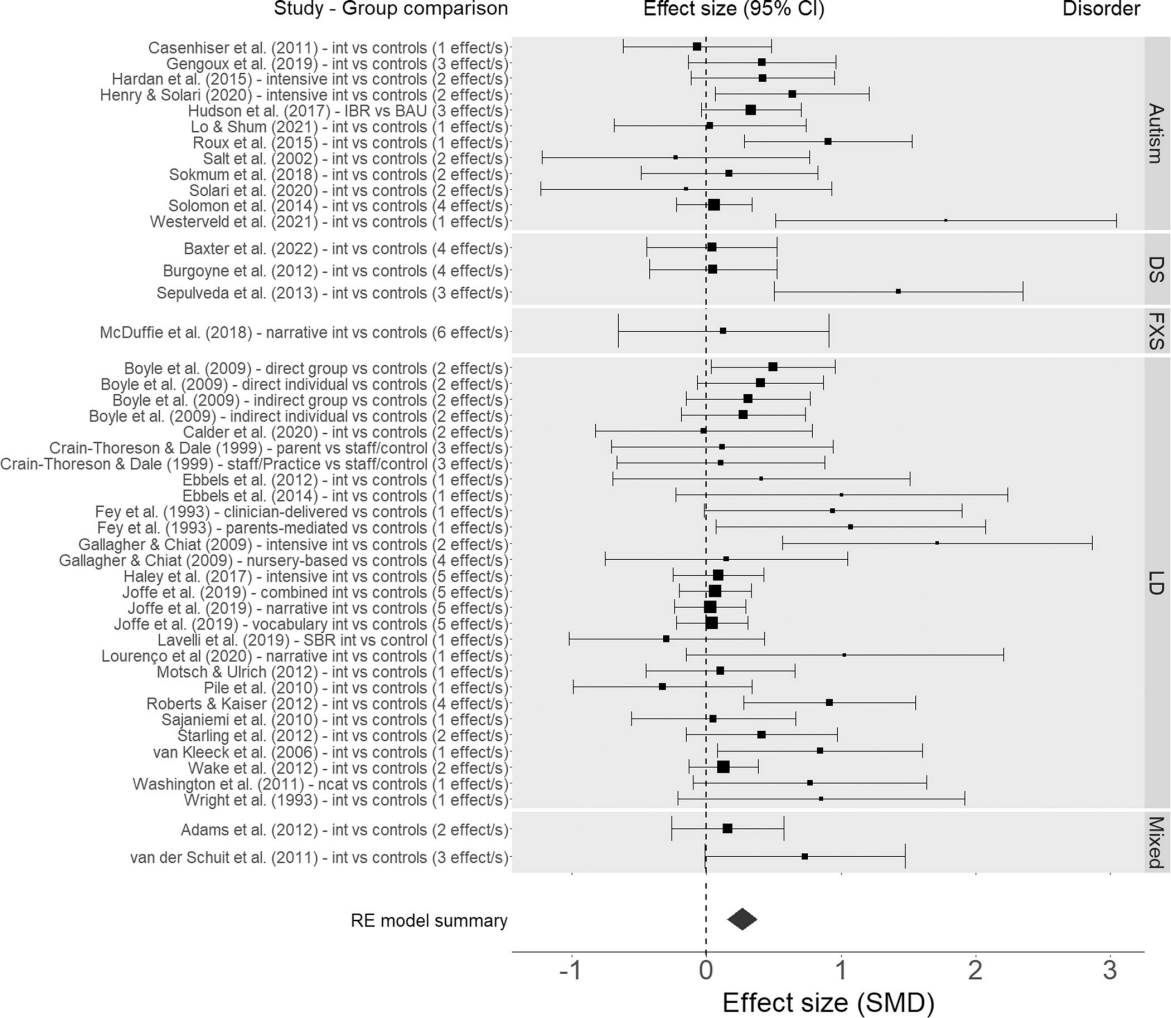
Forest plot of effects for pretest‐posttest comparisons aggregated by comparison‐within‐study. Error bars represent 95% confidence intervals (CIs). Larger dots indicate higher precision (i.e., smaller standard errors). The diamond below represents the overall meta‐analytic mean effect (its width represents the 95% CI).

Due to the large number of effect sizes, Figure 4 shows the forest plot of effect sizes aggregated by comparison‐within‐study (assuming a correlation between effects of 0.70), with information of the study and group comparisons in which they are clustered and group the studies by disorder. A sensitivity analysis based on all effect sizes used for the meta‐analysis was obtained by leaving one study out of the analysis at a time and recalculating the overall meta‐analytic effect with its 95% CI (see Supporting Information: Figure S1). The range of estimates was between 0.23 and 0.31. This was set to ensure that no single study had a major effect on the overall estimate. A second sensitivity analysis re‐included all studies regardless of the plausibility of their effect sizes. The estimated mean effect size was larger and much more uncertain, *d* = 0.35 [95% CI: 0.20, 0.50]. The overall heterogeneity was larger, *I* ^2^ = 70%. The leave‐one‐out range of variation was [0.32, 0.40].

Effect sizes for pre‐test/follow‐up comparisons were reported by eight studies, with 12 group comparisons and a total of 21 effects. The overall meta‐analytic estimate of the mean effect at follow‐up was *d* = 0.18 [95% CI: 0.03, 0.33]. (The follow‐up was conducted on average after 6 months, with a range of 3–12 months.) The estimated heterogeneity was small, total *I* ^2^ = 10%, with no heterogeneity estimated between studies, *τ*_study_ = 0.00, and minimal heterogeneity between group comparisons, *τ*_comparison_ = 0.06, and effect sizes, *ω* = 0.06. The forest plot of effect sizes at follow‐up aggregated by comparison‐within‐study (assuming a correlation between effects of 0.70) is reported in Supporting Information: Figure S2. Leave‐one‐out range of variation was [0.16, 0.23]. Sensitivity analysis conducted without excluding any (implausible) effect size, suggested a meta‐analytic estimate of *d* = 0.23 [95% CI: 0.06, 0.40], total *I* ^2^ = 28%, with a leave‐one‐out range of variation of [0.18, 0.29].

It should be noted that, for both main meta‐analytic models, heterogeneity was estimated as null at the between‐study level. Although the models correctly converged, this might be due to imperfect estimation linked with a complex data structure associated with a not so large number of observations. Forest plots in Figure 4 and Supporting Information: Figure S3, however, suggest that this might also be explained by the data: many studies present very large uncertainty bounds, meaning that their deviation from the overall effect might well be explained by sampling error. Furthermore, some of the most clearly heterogeneous effect sizes are indeed reported within the same study or group comparison (e.g., Boyle et al., 2009; Joffe et al., 2019; van der Schuit et al., 2011).

#### 5.3.2 Assessment of publication bias

**Figure 5:**
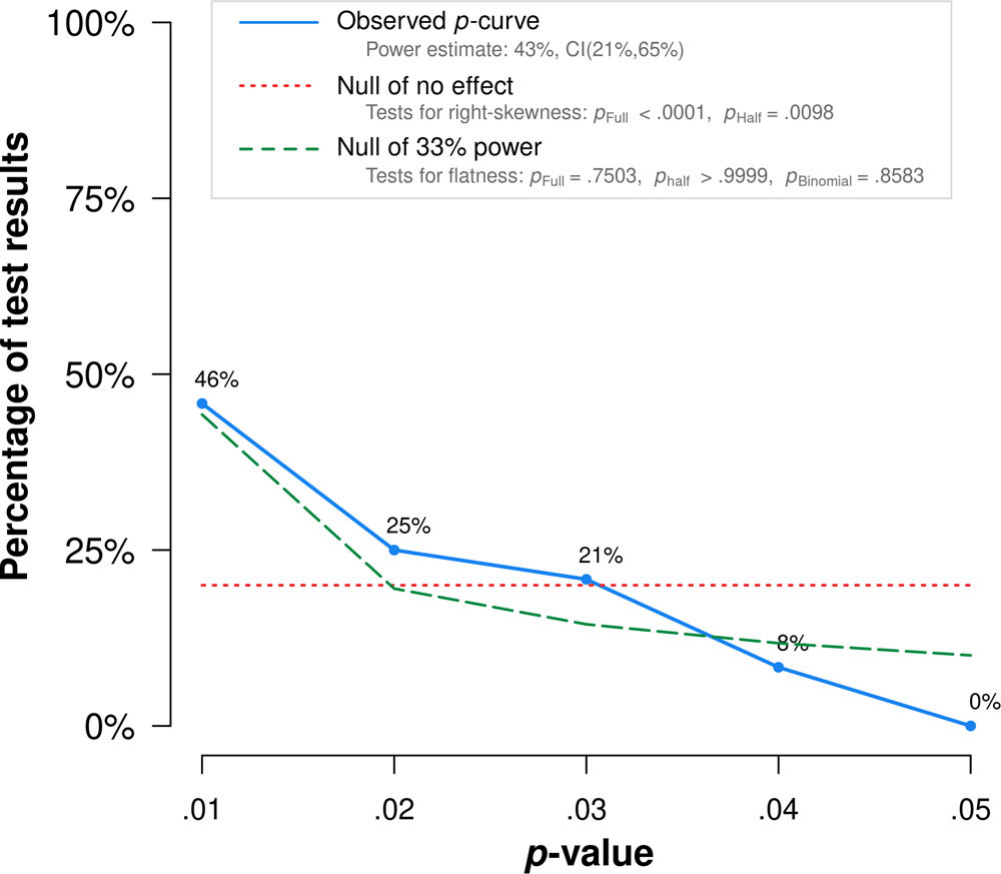
*P*‐curve (Simonsohn et al., 2014) calculated via online app.

Because of the limited number of different studies and effect sizes, the assessment of publication bias via *p*‐curve could be meaningfully conducted only for the pre‐test/post‐test comparison. Figure 5 shows the *p*‐curve analysis, which suggested no risk of publication bias at the level of the entire set of effect sizes. It included 24 statistically significant effects. The distribution of the significant *p*‐values was significantly right‐skewed (*p* = 0.0001), suggesting no anomalous concentration of *p*‐values around .05. The estimated power, based on the distribution of significant effects, was 43% [21%, 65%]. It should be noted that this analysis was based on *p*‐values recalculated from our z scores (not those reported directly by the studies); it did not consider the structure of dependencies among effects, and it could perform poorly as a result of between‐study heterogeneity (see the Methods section).

**Figure 6:**
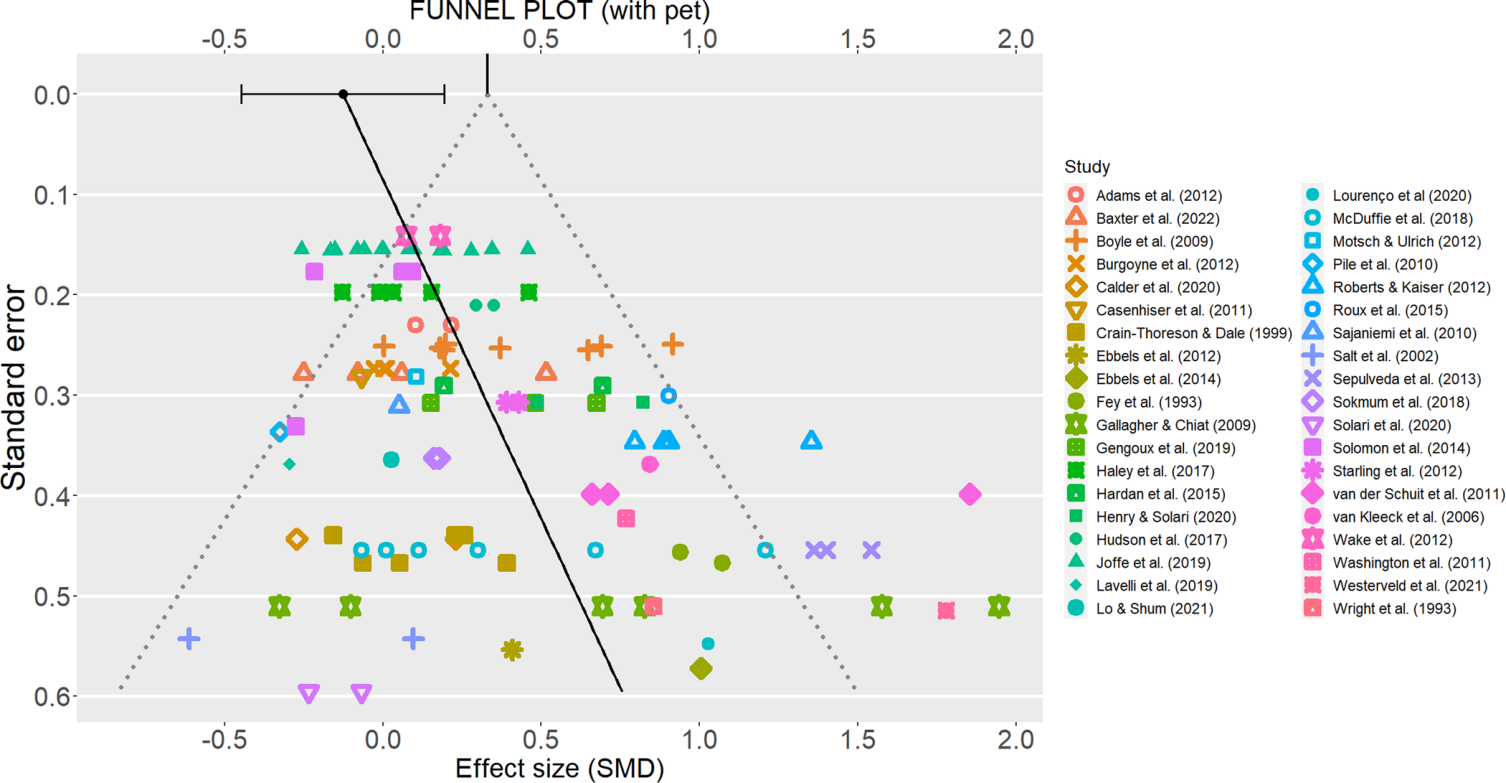
Funnel plot of all effect sizes (visually clustered by study), with PET meta‐regression slope and PET‐corrected effect size (dot on the top, with error bar representing its 95% confidence interval).

We also examined publication bias via the PET‐PEESE meta‐regression method. This approach allows us to model the structure of the dependencies among effect sizes and provides a bias‐free estimate of the effect size. We used an alternate set of variances estimated from the sample size alone to make them independent from effect sizes, but using the same meta‐analytic model presented above. For pre‐test/post‐test comparisons, the PET meta‐regression suggested that standard error was a significant positive moderator of the effect size, *B* = 1.48 [95% CI: 0.49, 2.47], *p* = 0.004. In other words, small studies tended to yield bigger effect sizes. The estimated bias‐corrected effect size (i.e., the intercept) was no longer significant and even became negative, *d* = −0.13 [95% CI: −0.45, 0.19], *p* = 0.439. An excess of bias correction is a known possibility in PET meta‐regression (Stanley & Doucouloagos, 2014). However, the PET‐PEESE method suggests that there is publication bias and that the bias‐corrected effect may be null. The funnel plot with the PET meta‐regression is shown in Figure 6. For pre‐test/follow‐up comparisons, the PET meta‐regression suggested that standard error was a statistically significant positive moderator of the effect size, *B* = 2.57, *p* = 0.020. Once again, the estimated bias‐corrected effect size was no longer statistically significant and it was even negative, *d* = −0.34 [−0.82, 0.13], *p* = 0.159. The funnel plot with the PET meta‐regression is shown in the Supporting Information: Figure S3.

#### 5.3.3 Analysis of moderators

Because of the limited number of studies, moderator analyses could be conducted only for pre‐test/post‐test comparisons.

##### Participants’ characteristics

There were two diagnostic conditions that had at least five studies—namely, language disorder (20 studies) and autism (12 studies). The moderator analysis showed no evidence of a difference between the two levels of the moderators, *Q*(1) = 0.13, *p* = 0.716, as the estimates for language disorder, *d* = 0.24 [95% CI: 0.09, 0.39], and autism, *d* = 0.29 [95% CI: 0.09, 0.48], were very similar.

There were enough studies for both “Clinical” (33 studies) and “Non‐clinical” (five studies) diagnostic status. The moderator analysis failed to suggest statistical significance, *Q*(1) = 3.28, *p* = 0.070, although the effect size appeared larger for “Clinical status,” *d* = 0.31 [95% CI: 0.19, 0.44] than for “Non‐clinical” status, *d* = 0.11 [95% CI: −0.07, 0.29].

All studies reported information on sample age; however, age was not a statistically significant moderator of the effect size, *Q*(1) = 0.016, *p* = 0.901 *B* = −0.0002. Twenty‐five studies included information on sex composition of the sample (proportion of boys), which did not moderate the effect size *Q*(1) = 0.16, *p* = 0.689, *B* = 0.17. Fourteen studies reported information about non‐verbal IQ and 10 reported language level at baseline. The level of non‐verbal IQ did not moderate effect size, *Q*(1) = 0.00, *p* = 0.985, *B* = 0.000. Language level did moderate effect size either, *Q*(1) = 1.49, *p* = 0.220, *B* = −0.02.

##### Components and implementation of language interventions

The intervention type had all three levels with sufficient study numbers for the moderator analysis; these levels were “explicit instruction” (21 studies), “implicit/incidental approach” (10 studies), and “hybrid approaches” (eight studies). The moderator analysis failed to show evidence of a difference among the three levels of the variable, *Q*(2) = 0.02, *p* = 0.988, with interventions on explicit instruction, *d* = 0.26 [95% CI: 0.12, 0.40], implicit/incidental approach, *d* = 0.25 [95% CI: 0.02, 0.49], and hybrid approaches, *d* = 0.28 [95% CI: 0.03, 0.53], showing relatively similar effects. It should be noted that the “implicit approach” was associated with participants being younger (*M*_age_ = 59.39 months) than in “explicit instruction” (*M*_age_ = 86.92 months) or “hybrid approach” (*M*_age_ = 79.97 months). However, age itself did not moderate the effect size (see above), and controlling for age left the effect of intervention type non‐significant and practically irrelevant, *Q*(2) = 0.03, *p* = 0.983.

As for the focus of the intervention, five levels of the moderator had an adequate number of studies for the analysis: vocabulary (six studies), grammar (six studies), multi‐component (seven studies), general language stimulation (eight studies), and book reading related programmes (eight studies). The moderator analysis did not reach significance, *Q*(4) = 2.71, *p* = 0.607. However, the failure to reach significance may have been due to lack of power, as the estimated effect sizes for different intervention foci were largely heterogeneous, giving the following results: vocabulary, *d* = 0.17 [95% CI: −0.13, 0.46]; grammar, *d* = 0.54 [95% CI: 0.16, 0.91]; multi‐component, *d* = 0.29 [95% CI: 0.05, 0.52]; general language stimulation, *d* = 0.36 [95% CI: 0.10, 0.62]; and book reading–related programmes, *d* = 0.22 [95% CI: −0.09, 0.53].

As for the language outcome measures, expressive and receptive vocabulary (15 and six studies, respectively), grammar (15 studies), expressive and receptive discourse (14 and 11 studies, respectively), and omnibus expressive and receptive tests (five studies each), had enough studies. This variable emerged as a statistically significant moderator of the effect size, *Q*(6) = 28.89, *p* < 0.001. The differences concerned receptive vocabulary and omnibus receptive tests, which showed smaller effect sizes, while the other outcomes presented rather homogeneous effects. For expressive vocabulary, *d* = 0.24 [95% CI: 0.09, 0.39]; for receptive vocabulary, *d* = 0.05 [95% CI: −0.12, 0.22]; for grammar, *d* = 0.31 [95% CI: 0.14, 0.48]; for expressive discourse, *d* = 0.34 [95% CI: 0.19, 0.48]; for receptive discourse, *d* = 0.45 [95% CI: 0.28, 0.62]; for omnibus expressive, *d* = 0.33 [95% CI: 0.11, 0.55]; for omnibus receptive measures, *d* = −0.04 [95% CI: −0.28, 0.20].

The type of setting revealed an adequate number of studies for interventions conducted at clinics (11 studies), children's homes (11 studies), and schools (15 studies). The test for moderators failed to reach the conventional threshold for statistical significance, *Q*(2) = 5.92, *p* = 0.052, but once again, this may have been due to a lack of power, as the estimated effect size for interventions conducted at the clinic, *d* = 0.55 [95% CI: 0.29, 0.81], was quite different from the estimates for the interventions conducted at children's homes, *d* = 0.21 [95% CI: 0.03, 0.38], and schools, *d* = 0.21 [95% CI: 0.08, 0.34]. We then examined delivery agents as a moderator of the effect sizes, including the clinicians (14 studies), teacher‐mediated (eight studies), and parent‐mediated (14 studies) categories, which had an adequate number of studies for the analysis. The moderator analysis did not reach significance for this factor, *Q*(2) = 1.33, *p* = 0.515. The effect sizes for interventions delivered by clinicians were *d* = 0.33 [95% CI: 0.12, 0.54], and those for parent‐mediated and teacher‐mediated interventions were *d* = 0.22 [95% CI: 0.05, 0.39] and *d* = 0.17 [95% CI: 0.00, 0.34], respectively.

We examined whether the session structure of the intervention and dosage explained heterogeneity across studies. As for the session structure of the intervention, there were enough studies for the individual and group categories (23 and 12 studies, respectively). The moderator analysis was not statistically significant, *Q*(1) = 0.835, *p* = 0.361, indicating that the effect size estimated for individual sessions, *d* = 0.29 [95% CI: 0.16, 0.43], and group sessions, *d* = 0.20 [0.03, 0.36], were similar. As for dosage, we examined session duration (23 studies), number of weeks of intervention (25 studies), and the total number of sessions (23 studies). We found that session duration and the number of weeks of intervention significantly moderated the effect size estimate. For session duration, *Q*(1) = 13.09, *p* = <0.001, *B* = 0.006; this means that, for every 15 min more of session duration, the effect size is estimated to increase by approximately *Δd* = 0.09. For the number of weeks of intervention, *Q*(1) = 5.75, *p* = 0.017, *B* = 0.027; this means that for every additional month of intervention, the effect size is estimated to increase by approximately *Δd* = 0.12. In contrast, the total number of sessions was not a statistically significant moderator of the treatment effect, *Q*(1) = 0.40, *p* = 0.530, *B* = 0.002.

##### Indicators of study quality

Recruitment had an adequate number of studies for four categories—namely, specialised centres (12 studies), special schools (10 studies), schools (six studies), and local agencies and advertising (five studies). The moderator analysis did not show a difference across the levels of the moderator, *Q*(3) = 1.334, *p* = 0.719. The effect sizes for specialised centres, *d* = 0.17 [95% CI: −0.02, 0.36], special schools, *d* = 0.27 [95% CI: 0.05, 0.50], schools, *d* = 0.21 [95% CI: −0.03, 0.39], and local agencies, *d* = 0.36 [95% CI: 0.09, 0.64], were indeed similar, although not identical.

As for the type of study design, we examined RCTs (17 studies) and QE interventions (21 studies). The moderator analysis was not statistically significant, although not far from it, *Q*(1) = 3.28, *p* = 0.070, and the estimates for QE studies, *d* = 0.40 [0.21, 0.60], and RCTs, *d* = 0.19 [0.07, 0.31], were quite divergent. As for the status of the control group, only studies using waiting lists (16 studies) and BAU conditions (19 studies) were sufficient to perform the analysis. In a similar fashion to the type of study design, the status of the control group did not reach significance, *Q*(1) = 2.39, *p* = 0.122. Nonetheless, the effect sizes for the waiting list, *d* = 0.17 [0.00, 0.33], and BAU, *d* = 0.35 [0.19, 0.50], were quite divergent. We then examined the type of language tests, including “standardised tests” (29 studies), “observational measures” (seven studies) and parent‐report (five studies), which all had a sufficient number of studies for the analysis. These variables did not moderate the overall effect size estimate, *Q*(2) = 0.57, *p* = 0.752, with standardised tests, *d* = 0.22 [0.11, 0.33], observational measures, *d* = 0.33 [0.05, 0.62], and parent‐report questionnaires, *d* = 0.25 [0.00, 0.51], showing similar effect sizes.

We considered country as a possible moderator and the Europe (17 studies), the US (10 studies) and other (11 studies) categories for the analysis, which represented enough studies to be reported. These variables did not moderate the effect size, *Q*(2) = 2.18, *p* = 0.336. The estimated effect sizes were as follows: Europe, *d* = 0.19 [0.04, 0.33]; the US, *d* = 0.36 [0.18, 0.55]; and other countries, *d* = 0.24 [0.02, 0.46]. Finally, we assessed the publication year and publication status. The former (38 studies) was not a statistically significant moderator of the effect size, *Q*(1) = 1.82, *p* = 0.177, *B* = −0.013, while the latter lacked unpublished studies to perform the analysis (all studies had been published).

#### 5.3.4 Retrospective consideration of power

Our meta‐analysis suggested that the true average (net) effect size for pre‐test/post‐test comparison is most likely between *d* = 0.15 and *d* = 0.38 (considering the 95% CI). The overall point estimate was *d* = 0.27. The overall heterogeneity was modest. As explained above, we adopted the simulation procedure and code offered by Toffalini et al. (2021) to perform power analysis for a pre‐test/post‐test‐control design. All simulations were run with 5000 iterations, assuming test/re‐test stability of *r* = 0.70 and a critical *α* = 0.05 for statistical significance. Assuming *d* = 0.15, a sufficient power of 80% would be reached with a total sample of *N* = 720 (i.e., *n* = 360 per group); assuming *d* = 0.38, the same power would be reached with a total sample of only *N* = 112 (i.e., *n* = 56 per group). However, for the most likely true effect size, *d* = 0.27, 80% power is achieved with *N* = 220 (i.e., *n* = 110 per group). Nonetheless, power could be increased even with the same sample size, using repeated measurements for each time point and/or using measures with higher reliability (see Toffalini et al., 2021).

Across the studies/group comparisons included in our meta‐analysis, the median sample size was *N* = 43 (about *n* = 22 per group). None of the group comparisons would reach the required level of power for *d* = 0.27, as indicated above (the largest sample size for a single group comparison was 200, including 99 treated individuals and 101 controls). Considering the median sample size of 43 (i.e., *n* = 22 per group), the median power of the studies that we reviewed could be estimated at 22% for *d* = 0.27; considering the uncertainty bounds, it could be estimated at 10% for *d* = 0.15 to 39% for *d* = 0.38. The exaggeration ratios (i.e., the ratio between the estimated effect size associated with *p* < 0.05 and the assumed true effect size; Gelman & Carlin, 2014) is 2.02 for the point estimate, and 3.47 to 1.53 for the uncertainty bounds, suggesting that when the median study finds a statistically significant effect, it is poised to substantially overestimate the true treatment effect.

It should be noted that the actual power might even be much lower than indicated above. This is because our assessment of publication bias via the PET‐PEESE method suggested that the true effect size may even drop to zero after correcting for publication bias.

## 6 DISCUSSION

### 6.1 Summary of main results

Overall, the current meta‐analytic review yielded the following important findings regarding the effects of oral language interventions from a transdiagnostic perspective.

#### 6.1.1 Are oral language interventions effective to attenuate language problems in children across different neurodevelopmental disorders?

There are initial indications that oral language interventions can yield moderate effects on language. The majority of the intervention studies eligible for our meta‐analytic review targeted children with language disorder and children with autism. Effect sizes of the effectiveness of oral language interventions across these two groups were similar, showing that interventions for these neurodevelopmental conditions had moderate effects in attenuating language problems at post‐test. Evidence on follow‐up evaluations, available for only eight studies, pointed to a smaller but still positive and statistically significant effects after an average of 6 months from the end of the intervention. However, this conclusion is tempered by evidence of publication bias. Publication bias analysis showed that results at post‐test and follow‐up were overestimated and that the true effect sizes were no longer significant after correcting for publication bias. Pre‐registration and replications of more robust and adequately powered trials—including a wider range of diagnostic conditions and more extensive reporting on potential moderator characteristics—are needed to drive evidence‐based practice and policy.

#### 6.1.2 What factors do moderate the response to treatment?

We tested the moderating role related to response to treatment by examining participant characteristics, components and implementation of the language interventions, and indicators of study quality. As for participants’ characteristics, children's diagnosis, diagnostic status, age, sex, non‐verbal cognitive ability and severity of language impairment at baseline were not statistically significant. The same occurred for moderators regarding the implementation of the language interventions, such as characteristics related to the intervention content, setting, delivery agent, session structure of the intervention or total number of sessions. However, language outcome measures and aspects of dosage were associated with variation in treatment outcomes. Receptive vocabulary and omnibus receptive tests showed smaller effect sizes compared to expressive vocabulary, grammar, expressive and receptive discourse, and omnibus expressive measures. In addition, longer sessions conducted over a longer period of time were more beneficial than brief sessions and short‐term interventions. Finally, indicators of study quality (recruitment, study design, status of control group, type of outcome test, country, and publication year) were not statistically significant moderators. Notably, the meta‐analysis had limited power to detect differences in the moderator analysis, largely because most studies to date have limited power to detect expected modest treatment effects.

Our findings align well with the meta‐analytic review by Rogde et al. (2019) on the effects from oral language interventions on standardised measures of language and reading comprehension. As for the language outcomes, Rogde et al. (2019) showed a mean effect of *d* = 0.16. As for earlier reviews in this area, for instance, Law et al. (2004) showed very large effects on vocabulary (*d* = 0.98) and grammar (*d* = 0.70), Cirrin and Gillam (2008) demonstrated also large effects on vocabulary (*d* between 0.5 and 3.5), Cirrin et al. (2010) also showed large effects on language measures but also a lot of variation between studies (effects ranging from *d* 0 to 1.65). Note that these studies focused on children with primary language difficulties and no other developmental concerns, and were concerned with delivery of intervention by qualified speech and language therapists. Overall, our review in general showed lower effects than previous reviews when different neurodevelopmental disorders are examined and publication bias is taken into account.

There can be several reasons for the discrepancy between our review and previous reviews. One apparent reason is that many of the previous reviews included studies with less well‐controlled designs than ours. It has been demonstrated that intervention studies that are less well‐controlled typically overestimate effects (Lipsey & Wilson, 1993). Thus, as study quality have been improved over the years towards more RCTs, it is also likely that we will experience a downward trend in effect sizes. Another issue that might explain this discrepancy is that many of the previous reviews code outcomes that are constructed by the researchers and contain elements from the training, together with standardised outcomes. Such research made, bespoke measures generally yield much larger effects than standardised measures (Rogde et al., 2021).

As for the moderator analysis, receptive vocabulary and omnibus receptive tests showed smaller effect sizes compared to expressive vocabulary, expressive language, and omnibus expressive measures, which is not surprising given previous studies of these kinds of language interventions. Typically at pre‐test, previous studies find that the different language tests constitute a global oral language construct. However, at post‐test, most studies do not realise measurement invariance, mainly because the training has affected the measures in the global language construct unevenly (e.g., see Hagen et al., 2017; Rogde et al., 2016; West, Snowling et al., 2021). Typically there are larger impacts from the training on expressive measures than on receptive measures (Hagen et al., 2017; Melby‐Lervåg et al., 2020; Rogde et al., 2016). Thus, these kinds of interventions do not seem to be able to improve the language construct per se, only aspects of it. However, this does not mean that the improvement is without value, expressive language is important and improving this might have positive repercussions, for example in the classroom or social participation. It also opens up the possibility that the improvement or some of the improvement might reflect that children receiving the intervention become less cautious and shy, and are better able to use their existing language in social settings. If this is the case, then the effects might not be caused by improvement of aspects of the language construct, but rather in children's willingness to use the language skills they have. Thus, in the context of active control groups (rather than BAU or passive control groups as here) the expressive effects might be reduced.

### 6.2 Overall completeness and applicability of evidence

The meta‐analytic review shows that there are no transdiagnostic studies or studies comparing treatment effects across diagnostic conditions. Unfortunately, we did not find oral language intervention studies on children with Williams syndrome that matched our inclusion criteria. Another challenge is the considerable variation in how language disorders are characterised and how diagnostic criteria are applied.

### 6.3 Quality of the evidence

The main problem with studies in the considered area is that most have an unclear risk of bias. This is because the reporting quality is generally poor and many studies fail to give the necessary levels of detail about the study design. For instance, most studies report that they have used a randomisation procedure, but few studies report on how the randomisation was carried out. This lack of detail in the reporting affects not only the randomisation but also the other risk of bias categories, such as allocation concealment, blinding, incomplete reporting and selective reporting. Despite unclear reporting in general, it is clear that most studies are underpowered. The median power of the studies that we reviewed could be estimated at 22% for *d* = 0.27. That many of them still report significant findings is probably due to a combination of small groups exhibiting spurious but large effects in the short term, compounded by publication bias. In these selected groups, the standard deviation on the outcome measures can often be small, and this will also deflate the effect when there are small mean differences (because the SD is the denominator in the Cohen's *d* calculation; Ingre, 2013). However, it should be noted that many studies featured non‐significant (combined) effect sizes (see Figure 4, where most CIs overlap with zero). However, it might still be that these were reported as significant in the paper because of the use of covariates. Moreover, it might be that many studies leverage individual significant tests without combining multiple outcomes or controlling for multiple comparisons.

### 6.4 Potential biases in the review process

First, due to staff changes, which consisted of E.T. and K.R. joining the team during the implementation of the meta‐analysis, different people were involved in doing the review versus the original protocol. This resulted in some changes in the review compared with the protocol. It also resulted in a long time to conduct the review and the analyses after the protocol was written. However, the search was updated, so this should not create any bias. Second, several studies were excluded because of insufficient data to calculate the effect size. Although we did send emails to authors, the response rate was low, and this might have created a bias. Third, determining what is actually a “language” intervention was not straightforward. We excluded many studies, particularly those aimed at children with autism, that had a primary focus on social skills and where language was one of several different outcomes but not the primary (or named secondary) outcome measure. Instead, we included only those studies that were explicit in their descriptions of the intervention or outcomes as focusing on developing oral language. Fourth, we focused on oral language interventions and oral language outcomes, given the associations of oral language with education, health, and social outcomes. However, we note that for many children with neurodevelopmental disorders, oral language may not be a suitable or desirable goal of intervention. We did not feel it would be appropriate to compare oral language interventions with those that focus on augmentative and alternative communication (AAC), but we acknowledge that for many young people with neurodevelopmental disorders AAC would be a valid and appropriate intervention focus (see O'Neill et al., 2018 for a relevant meta‐analysis). Fifth, it was not possible to include all neurodevelopmental disorders outlined in the pre‐registration because there were no group intervention studies meeting our criteria for some conditions. While single case studies may exist for these conditions, we made an a priori decision not to include single case designs in this review though these designs may provide additional clinical insights. As for the moderator analysis, we did not examine some potentially relevant variables, such as socioeconomic status or maternal education, in part because this was not reliably reported in the papers. In addition, we stress that the moderator analyses are not causal. Thus, this means that there could be other third variables underlying, for instance, the differences between two sets of studies. Therefore, our findings and recommendations should be interpreted with caution, since the samples have not been randomised on the moderators. In addition, it should be noted that for some moderators, there were few studies; therefore, important relationships might have been difficult to detect. Finally, we lacked access to Education Source (EBSCO), British Education Index (EBSCO), Academic Search Complete (EBSCO), and ProQuest Digital Dissertations databases, listed in our study pre‐registration. While most records in the first three databases have likely been identified with others consulted for this meta‐analysis, we might have missed detecting dissertations that could have been potentially relevant to the scope of this review.

### 6.5 Agreements and disagreements with other studies or reviews

See the discussion section for an in detail account of this.

## 7 AUTHORS’ CONCLUSIONS

### 7.1 Implications for practice

Our meta‐analytic review has implications for practice and policy. The current meta‐analysis points out that a transdiagnostic perspective might be a fruitful approach to planning and delivering interventions, as we did not find significant differences in treatment effects for different neurodevelopmental disorders (but note that not all diagnostic conditions were included). This is particularly important for health and education systems organised around diagnosis‐specific care pathways. The evidence presented in this review indicates that condition‐specific language interventions are not necessarily required. Providing intervention to mixed groups or caseloads of children could be more cost‐effective, representing an important question for future research. For most children with neurodevelopmental disorders, continuous and longer‐term support is needed. It is therefore noteworthy that the effects from the shorter interventions that characterise most of these studies are not sufficient to ameliorate child language problems in the long run, as shown in the few studies that did report follow‐up measures. As for moderators, age and non‐verbal ability did not moderate intervention effects; thus, older children and children with intellectual disabilities may well benefit from focused language intervention. Not only is early intervention important, but later interventions can make a difference. It is important to note that how the children were assessed before the intervention might play a role in the effects of the interventions. Groups with clinical diagnoses reported higher effects than intervention for children identified to have language difficulties on a screening measure. One reason for this could be that samples with difficulties were more heterogeneous but the interventions were rather standardised and “targeted” interventions, meaning that they were less likely to be adapted to individual learning needs. However, it should be noted that children with autism and genetic conditions are more likely to be included in the clinical diagnosis group, whereas the groups with “difficulties” mainly include children with low language proficiency that may or may not meet clinical diagnostic criteria.

### 7.2 Implications for research

Based on our analysis of study quality, several different recommendations can be highlighted for future studies. First of all, the results showed indications of considerable publication bias in the results. To handle this, it is critical to pre‐register future studies to ensure that null findings are reported. Second, we noted that many of the included trials were conducted in special schools or clinics by clinicians, who may have a vested interest in demonstrating positive treatment outcomes. These conflicts of interest may contribute to publication bias and should be noted more explicitly in published trials. Third, most studies were severely underpowered. Since the groups in question here are low‐incidence populations, for future studies, it is critical to have cross‐laboratory and cross‐country collaborations to replicate findings and increase sample sizes. We encourage clinical researchers to collaborate with statisticians in a priori power analyses and in designing trials and analysing intervention outcomes. In addition, using a transdiagnostic approach might make it easier to conduct more adequately powered studies. Using such an approach is also more akin to the reality of clinical and educational caseloads. Fourth, many studies did not report vital information; in particular, details about randomisation, attrition and quality of the outcome measures were often missing. To ensure that needed information is present, future studies should use a standardised way of reporting intervention studies, such as standard reporting of participant characteristics and intervention parameters. Few studies were pre‐registered and this will be important to change. Fifth, the studies used a large variety of outcome measures. There is a need for harmonisation of language assessments to facilitate data quality and data sharing in future studies. In medicine, different areas have developed tools that list preferred outcomes based on their validity and utility. It would be a great advantage to have something similar in the area of language, as this would clearly have the potential to improve research. In addition, only eight studies reported follow‐up effects, and out of those, none looked at effects beyond 12 months. There is a need for more long‐term follow‐up, as well as interventions that are longer lasting than the average 6 months that was the case here. We know from many studies that intervention effects tend to fade out rather quickly (Bailey, Duncan, Cunha, et al., 2020; Bailey, Duncan, Odger, et al., 2017), and determining how to prevent fade‐out should be an important focus in future studies. Finally, none of the studies discuss adverse effects from these interventions, for instance, in relation to the fact that the children were often taken out of the classroom and that the interventions often took away time that could have been used in a different way. In future studies, it could also be important to include this perspective on adverse effects, and if possible, measure such effects. More knowledge about interventions for children with neurodevelopmental disorders is critical because this is an issue that most school systems and healthcare systems across the world are struggling to address. However, intervention research targeting these groups seems immature. In future studies, it is important to follow the above recommendations to develop more robust knowledge about interventions that can be used to develop better educational and clinical support for children with neurodevelopmental disorders.

## CONTRIBUTIONS OF AUTHORS

Content: Donolato, Toffalini, Rogde, Norbury, Nordahl‐Hansen, Lervåg and Melby‐Lervåg

Systematic review methods: Donolato, Toffalini, Rogde, Norbury, Nordahl‐Hansen, Lervåg and Melby‐Lervåg

Statistical analysis: Donolato, Toffalini, Lervåg and Melby‐Lervåg

Information retrieval: Donolato and Melby‐Lervåg collaborated with information retrieval experts at the library of the University of Oslo

## DECLARATIONS OF INTEREST

The authors have no conflict of interest to declare.

## PLANS FOR UPDATING THIS REVIEW

The team will be responsible for updating every 10 years.

## DIFFERENCES BETWEEN PROTOCOL AND REVIEW

Some deviations from the protocol were conducted in the review process:

- Search: Education Source (EBSCO), British Education Index (EBSCO), Academic Search Complete (EBSCO) and ProQuest Digital Dissertations were not used in the literature search, as the University of Oslo does not have access to these databases.
- Participants: We included studies that used at least the 16th percentile (−1 SD) on standardised tests to identify children with language difficulties. In the protocol, we did not impose an a priori cut‐off for the level or profile of language deficit required for inclusion in this review.
- Moderators: We added country as a moderator to account for this variable in the analysis. We also removed Sample size as a moderator because we performed the PET‐PEESE analysis for publication bias; this analysis applies a correction of the estimates accounting for studies with small samples.
- Analysis: We decided to check extreme values to ensure that the effect sizes for the analysis were plausible and that the intervention and control groups were matched at the baseline for the outcomes evaluating the efficacy of the intervention. We did not perform meta‐analytic structural equation modelling (MASEM) because the number of studies prevented us from conducting this analysis. As for the assessment of publication bias, we added PET‐PEESE to evaluate the consistency of the results.
- Risk of bias assessment: We conducted the study appraisal to evaluate the methodological quality of the selected studies with the Cochrane Collaboration's scale for assessing risk of bias in randomised trials (Higgins et al., 2011) instead of the Grading of Recommendations, Assessment, Development and Evaluation system (GRADE; Guyatt et al., 2008).

## SOURCES OF SUPPORT

**Internal sources**

- No sources of support provided

**External sources**

- FINNUT grant (237724), Research Council Norway, to Monica Melby‐Lervåg (Principal Investigator), Norway
- Economic and Social Research Council (ES/R003041/1) grants to Courtenay Norbury (Principal Investigator), UK
- FINNUT grant (283586), Research Council Norway, to Arne Lervåg (Principal Investigator), Norway

## Supporting information

Supporting information.
