# Correction to “Oral language interventions can improve language outcomes in children with neurodevelopmental disorders: A systematic review and meta‐analysis”

**DOI:** 10.1002/cl2.1391

**Published:** 2024-03-11

Donolato, E., Toffalini, E., Rogde, K., Nordahl‐Hansen, A., Lervåg, A., Norbury, C., & Melby‐Lervåg, M. (2023). Oral language interventions can improve language outcomes in children with neurodevelopmental disorders: A systematic review and meta‐analysis. *Campbell Systematic Reviews*, 19(4), e1368.

Affiliations of three of the authors were incorrect and the correct affiliations should read:

Enrica Donolato^1^ | Enrico Toffalini^2^ | Kristin Rogde^3^ | Anders Nordahl‐Hansen^4^ | Arne Lervåg^1,5^ | Courtenay Norbury^3,6^ | Monica Melby‐Lervåg^3,5^

^1^Department of Education, University of Oslo, Oslo, Norway

^2^Department of General Psychology, University of Padova, Padova, Italy

^3^Department of Special Needs Education, University of Oslo, Oslo, Norway

^4^Department of Education, ICT and Learning, Østfold University College, Halden, Norway

^5^Centre for Research on Equality in Education (CREATE), University of Oslo, Oslo, Norway

^6^Division of Psychology & Language Sciences, University College London, London, UK

We apologize for this error.

