# Supplementary material for: Oral language interventions can improve language outcomes in children with neurodevelopmental disorders: A systematic review and meta‐analysis
Source: Campbell Syst Rev. 2023 Nov 27;19(4):e1368. doi: 10.1002/cl2.1368 (PMC10680434; doi:10.1002/cl2.1368)

# Online Supplement 1: Database Search Strategy

| **Database** | **#** | **Searches** |
| --- | --- | --- |
| **Embase, ERIC, Ovid MEDLINE(R), APA PsycINFO** | 1 | (((Intellectual or developmental) adj2 (disabili* or disorder* or delay*)) or (Mental adj2 retard*) or Specific Language Impair* or SLI or ((Developmental Language or Semantic pragmatic or Expressive language or Pervasive development*) adj2 disorder*) or (Language adj2 (Delay* or defic* or impair* or problem* or disorder*)) or PDD or PDD NOS or Autis* or Asperger or ASD or Trisomy 21 or (Chromosome adj1 "21") or (mongol or mongols or mongoloid or mongolism or mongolianism) or Fragile X or FXS or (Fragile adj1 X) or (Chromosome adj1 X) or ((Down* or Marker X or Martin Bell or Williams or Williams Beuren) adj2 (disease* or syndrome)) or WS).tw,kw,kf. |
|  | 2 | (exp Intellectual Disability/ or exp Language Development Disorders/ or Developmental Disabilities/ or exp Neurodevelopmental Disorders/ or exp Language Disorders/ or exp Child Development Disorders, Pervasive/ or Down Syndrome/ or Fragile X Syndrome/ or Williams Syndrome/) use medall |
|  | 3 | (mental deficiency/ or down syndrome/ or developmental disorder/ or developmental delay/ or exp language disability/ or exp developmental language disorder/ or autism/ or asperger syndrome/ or "pervasive developmental disorder not otherwise specified"/ or Down syndrome/ or fragile X syndrome/ or Williams Beuren syndrome/) use emczd |
|  | 4 | (intellectual disability/ or down syndrome/ or mild intellectual disability/ or moderate intellectual disability/ or severe intellectual disability/ or learning disabilities/ or exp Intellectual Disability/ or exp language impairments/ or pervasive developmental disorders/ or asperger syndrome/ or autism/ or developmental disabilities/ or down syndrome/) use psyh |
|  | 5 | (intellectual disability/ or down syndrome/ or mild intellectual disability/ or moderate intellectual disability/ or severe intellectual disability/ or developmental disabilities/ or pervasive developmental disorders/ or language impairments/ or asperger syndrome/ or autism/ or down syndrome/) use eric |
|  | 6 | or/1-5 |
|  | 7 | (language adj3 (intervent* or treat* or educat* or therap* or train* or facilit* or program*)).tw,kw,kf. |
|  | 8 | Language Therapy/ use medall |
|  | 9 | (language therapy/ or (exp Intervention/ and exp Language/)) use psyh |
|  | 10 | language therapy/ use emczd |
|  | 11 | (exp Intervention/ and exp Language/) use eric |
|  | 12 | or/7-11 |
|  | 13 | (Vocabulary or (Word adj3 (knowledge or learn*)) or ((Linguistic or language or listening) adj3 comprehen*) or (Language adj3 (proficien* or skill* or abilit* or acquisition or development*)) or ((Oral or spoken) adj2 language) or Gramma* or Syntax or Syntactic* or Semantic* or Morph* or (Narrative adj (skill* or comprehen*)) or narration or (Figurative adj3 (speech or language or pragmatic*))).tw,kf,kw. |
|  | 14 | (Vocabulary/ or Paired-Associate Learning/ or Language Tests/ or Language Arts/ or Language Development/ or exp Linguistics/ or Semantics/) use medall |
|  | 15 | (exp vocabulary/ or figurative language/ or "morphology (language)"/ or Semantics/ or exp Syntax/ or Narratives/ or language proficiency/) use psyh |
|  | 16 | (exp vocabulary/ or exp Language Acquisition/ or language proficiency/ or language skills/ or oral language/ or grammar/ or "morphology (languages)"/ or syntax/ or semantics/ or narration/ or figurative language/) use eric |
|  | 17 | (vocabulary/ or ((language/ or linguistics/) and comprehension/) or language ability/ or language/ or comprehension/ or linguistics/ or language ability/ or grammar/ or semantics/ or semantics/) use emczd |
|  | 18 | or/13-17 |
|  | 19 | (experiment* or randomi?ed or randomly or RCT or trial or group* or control or placebo).tw,kw,kf. |
|  | 20 | (randomized controlled trial or controlled clinical trial).pt. use medall |
|  | 21 | (exp Clinical trial/ or Randomized controlled trial/) use emczd |
|  | 22 | randomized controlled trials/ use eric |
|  | 23 | randomized controlled trials/ use psyh |
|  | 24 | 6 and 12 and 18 and 18 |
|  | 25 | 24 use medall |
|  | 26 | 24 use eric |
|  | 27 | 24 use psyh |
|  | 28 | 24 use emczd |
| **Cochrane Library (including ClinicalTrials.gov)** | #1 | (((Intellectual or developmental) NEAR/2 (disabili* or disorder* or delay*)) or (Mental NEAR/2 retard*) or Specific Language Impair* or SLI or ((Developmental Language or Semantic pragmatic or Expressive language or Pervasive development*) NEAR/2 disorder*) or (Language NEAR/2 (Delay* or defic* or impair* or problem* or disorder*)) or PDD or PDD NOS or Autis* or Asperger or ASD or Trisomy 21 or (Chromosome NEAR/1 "21") or (mongol or mongols or mongoloid or mongolism or mongolianism) or Fragile X or FXS or (Fragile NEAR/1 X) or (Chromosome NEAR/1 X) or ((Down* or Marker X or Martin Bell or Williams or Williams Beuren) NEAR/2 (disease* or syndrome)) or WS):ti,ab,kw |
|  | #2 | ([mh "Intellectual Disability"] or [mh "Language Development Disorders"] or [mh "Developmental Disabilities"] or [mh "Neurodevelopmental Disorders"] or [mh "Language Disorders"] or [mh "Child Development Disorders"] or [mh Pervasive] or [mh "Down Syndrome"] or [mh "Fragile X Syndrome"] or [mh "Williams Syndrome"]) |
|  | #3 | #1 or #2 |
|  | #4 | (language NEAR/3 (intervent* or treat* or educat* or therap* or train* or facilit* or program* or acquisition)):ti,ab,kw |
|  | #5 | [mh "Language Therapy"] |
|  | #6 | #4 OR #5 |
|  | #7 | (Vocabulary or (Word NEAR/3 (knowledge or learn*)) or ((Linguistic or language or listening) NEAR/3 comprehen*) or (Language NEAR/2 (proficien* or skill* or abilit* or development*)) or ((Oral or spoken) NEAR/2 language) or Gramma* or Syntax or Syntactic* or Semantic* or Morph* or (Narrative adj (skill* or comprehen*)) or narration or (Figurative NEAR/3 (speech or language or pragmatic*))):ti,ab,kw |
|  | #8 | ([mh Vocabulary] or [mh "Paired-Associate Learning"] or [mh "Language Tests"] or [mh "Language Arts"] or [mh "Language Development"] or [mh Linguistics] or [mh Semantics]) |
|  | #9 | #7 OR #8 |
|  | #10 | (experiment* or randomi?ed or randomly or RCT or trial or group* or control or placebo):ti,ab,kw |
|  | #11 | (randomized controlled trial or controlled clinical trial):pt |
|  | #12 | #10 OR #11 |
|  | #13 | #3 AND #6 AND #9 AND #12 |
| **Web of Science** | # 5 | #4 AND #3 AND #2 AND #1  Indexes=SCI-EXPANDED, SSCI, A&HCI, CPCI-S, CPCI-SSH, ESCI Timespan=All years |
|  | # 4 | TS=( (down disease*) OR ((mongol or mongols or mongoloid or mongolism or mongolianism) ) OR (fragile x) OR (fxs) OR (fragile NEAR/1 x) OR (chromosome NEAR/1 x) OR (marker x syndrome) OR (martin-bell syndrome) OR (williams syndrome) OR (william beuren syndrome) OR (intellectual disabilit*) OR (intellectual disorder*) OR (developmental disorder*) OR (development delay*) OR (development disabilit*) OR (mental NEAR/2 retard*) OR (language disorder) OR (specific language impair*) OR (SLI) OR (developmental language disorder) OR (language delay*) OR (language impair*) OR (language defic*) OR (language problem*) OR (semantic-pragmatic disorder*) OR (expressive language disorder*) OR (pervasive development* disorder*) OR (PDD) OR (PDD-NOS) OR (autis*) OR (asperger) OR (ASD) OR (down syndrome) OR (trisomy 21) OR (chromosome NEAR/1 21) )  Indexes=SCI-EXPANDED, SSCI, A&HCI, CPCI-S, CPCI-SSH, ESCI Timespan=All years |
|  | # 3 | TS=(Vocabulary or (Word NEAR/3 (knowledge or learn*) ) or ((Linguistic or language or listening) NEAR/3 comprehen*) or (Language NEAR (proficien* or skill* or abilit* or acquisition) ) or ((Oral or spoken) NEAR/2 language) or Gramma* or Syntax or Syntactic* or Semantic* or Morph* or (Narrative NEAR (skill* or comprehen*) ) or narration or (Figurative NEAR/3 (speech or language or pragmatic*) ))  Indexes=SCI-EXPANDED, SSCI, A&HCI, CPCI-S, CPCI-SSH, ESCI Timespan=All years |
|  | # 2 | TS=  (language NEAR/3 (intervent* or treat* or educat* or therap* or train* or facilit* or program* or acquisition) )  Indexes=SCI-EXPANDED, SSCI, A&HCI, CPCI-S, CPCI-SSH, ESCI Timespan=All years |
|  | # 1 | TS=  (experiment* or randomi?ed or randomly or RCT or trial or group* or control or placebo)  Indexes=SCI-EXPANDED, SSCI, A&HCI, CPCI-S, CPCI-SSH, ESCI Timespan=All years |
| **CINAHL (EBSCO)** | S1 | TI (((Intellectual or developmental) N2 (disabili* or disorder* or delay*)) or (Mental N2 retard*) or Specific Language Impair* or SLI or ((Developmental Language or Semantic pragmatic or Expressive language or Pervasive development*) N2 disorder*) or (Language N2 (Delay* or defic* or impair* or problem* or disorder*)) or PDD or PDD NOS or Autis* or Asperger or ASD or Trisomy 21 or (Chromosome N1 "21") or (mongol or mongols or mongoloid or mongolism or mongolianism) or Fragile X or FXS or (Fragile N1 X) or (Chromosome N1 X) or ((Down* or Marker X or Martin Bell or Williams or Williams Beuren) N2 (disease* or syndrome)) or WS) OR AB (((Intellectual or developmental) N2 (disabili* or disorder* or delay*)) or (Mental N2 retard*) or Specific Language Impair* or SLI or ((Developmental Language or Semantic pragmatic or Expressive language or Pervasive development*) N2 disorder*) or (Language N2 (Delay* or defic* or impair* or problem* or disorder*)) or PDD or PDD NOS or Autis* or Asperger or ASD or Trisomy 21 or (Chromosome N1 "21") or (mongol or mongols or mongoloid or mongolism or mongolianism) or Fragile X or FXS or (Fragile N1 X) or (Chromosome N1 X) or ((Down* or Marker X or Martin Bell or Williams or Williams Beuren) N2 (disease* or syndrome)) or WS) |
|  | S2 | (MH "Intellectual Disability+") OR (MH "Language Disorders+") OR (MH "Developmental Disabilities") OR (MH "Child Development Disorders, Pervasive+") OR (MH "Down Syndrome") OR (MH "Fragile X Syndrome") OR (MH "Williams Syndrome") |
|  | S3 | S1 OR S2 |
|  | S4 | TI (language N3 (intervent* or treat* or educat* or therap* or train* or facilit* or program*)) OR AB (language N3 (intervent* or treat* or educat* or therap* or train* or facilit* or program*)) |
|  | S5 | (MH "Language Therapy") |
|  | S6 | S4 OR S5 |
|  | S7 | TI (Vocabulary or (Word N3 (knowledge or learn*)) or ((Linguistic or language or listening) N3 comprehen*) or (Language N2 (proficien* or skill* or abilit* or acquisition or development*)) or ((Oral or spoken) N2 language) or Gramma* or Syntax or Syntactic* or Semantic* or Morph* or (Narrative N (skill* or comprehen*)) or narration or (Figurative N3 (speech or language or pragmatic*))) OR AB (Vocabulary or (Word N3 (knowledge or learn*)) or ((Linguistic or language or listening) N3 comprehen*) or (Language N2 (proficien* or skill* or abilit* or acquisition)) or ((Oral or spoken) N2 language) or Gramma* or Syntax or Syntactic* or Semantic* or Morph* or (Narrative N (skill* or comprehen*)) or narration or (Figurative N3 (speech or language or pragmatic*))) |
|  | S8 | (MH "Vocabulary") OR (MH "Language Tests+") OR (MH "Language Arts+") (MH "Language Development") OR (MH "Linguistics+") OR (MH "Semantics") |
|  | S9 | S7 OR S8 |
|  | S10 | TI (experiment* or randomi*ed or randomly or RCT or trial or group* or control or placebo) OR AB (experiment* or randomi*ed or randomly or RCT or trial or group* or control or placebo) |
|  | S11 | (MH "Randomized Controlled Trials+") |
|  | S12 | PT randomized controlled trial |
|  | S13 | S10 OR S11 OR S12 |
|  | S14 | S3 AND S6 AND S9 AND S13 |
| **Scopus** |  | ( TITLE-ABS-KEY ( ( language W/3 ( intervent* OR treat* OR educat* OR therap* OR train* OR facilit* OR program* OR acquisition ) ) ) ) AND ( TITLE-ABS-KEY ( ( vocabulary OR ( word W/3 ( knowledge OR learn* ) ) OR ( ( linguistic OR language OR listening ) W/3 comprehen* ) OR ( language W/1 ( proficien* OR skill* OR abilit* OR acquisition ) ) OR ( ( oral OR spoken ) W/2 language ) OR gramma* OR syntax OR syntactic* OR semantic* OR morph* OR ( narrative W/1 ( skill* OR comprehen* ) ) OR narration OR ( figurative W/3 ( speech OR language OR pragmatic* ) ) ) ) ) AND ( TITLE-ABS-KEY ( ( experiment* OR randomi?ed OR randomly OR rct OR trial OR group* OR control OR placebo ) ) ) AND ( TITLE-ABS-KEY ( ( ( intellectual OR developmental ) W/2 ( disabili* OR disorder* OR delay* ) ) OR ( mental W/2 retard* ) OR ( specific AND language AND impair* ) OR sli OR ( developmental AND language AND disorder ) OR ( semantic AND pragmatic AND disorder ) OR ( expressive AND language AND disorder ) OR ( pervasive AND development* AND disorder ) OR ( ( language ) W/2 ( delay* OR defic* OR impair* OR problem* OR disorder* ) ) OR pdd OR pdd-nos OR autis* OR asperger OR asd OR ( trisomy 21 ) OR ( chromosome W/1 21 ) OR mongol OR mongols OR mongoloid OR mongolism OR mongolianism OR "Fragile X" OR fxs OR ( fragile W/1 x ) OR ( chromosome W/1 x ) OR ( ( down* OR "Marker X" OR "Martin Bell" OR williams OR "Williams Beuren" ) W/2 ( disease* OR syndrome ) ) ) ) |
| **Epistemonikos** |  | (title:("Intellectual disabilit*" OR "Intellectual disorder*" OR "Developmental disorder*" OR "Developmental delay*" OR "Developmental disabilit*" OR "Mental retard*" OR "Language Disorder" OR "Specific Language Impair*" OR "SLI" OR "Developmental Language Disorder" OR "Language Delay*" OR "Language defic*" OR "Language impair*" OR "Language problem*" OR "Semantic‐pragmatic disorder*" OR "Expressive language disorder*" OR "Pervasive development* disorder*" OR "PDD" OR "PDD‐NOS" OR "Autis*" OR "Asperger" OR "ASD" OR "Down Syndrome" OR "Trisomy 21" OR "Chromosome 21" OR "Down* Disease") OR abstract:("Intellectual disabilit*" OR "Intellectual disorder*" OR "Developmental disorder*" OR "Developmental delay*" OR "Developmental disabilit*" OR "Mental retard*" OR "Language Disorder" OR "Specific Language Impair*" OR "SLI" OR "Developmental Language Disorder" OR "Language Delay*" OR "Language defic*" OR "Language impair*" OR "Language problem*" OR "Semantic‐pragmatic disorder*" OR "Expressive language disorder*" OR "Pervasive development* disorder*" OR "PDD" OR "PDD‐NOS" OR "Autis*" OR "Asperger" OR "ASD" OR "Down Syndrome" OR "Trisomy 21" OR "Chromosome 21" OR "Down* Disease")) AND (title:(("language intervent*" OR "language treat*" OR "language educat*" OR "language therap*" OR "language train*" OR "language facilit*" OR "language program*" OR "language acquisition")) OR abstract:(("language intervent*" OR "language treat*" OR "language educat*" OR "language therap*" OR "language train*" OR "language facilit*" OR "language program*" OR "language acquisition"))) |
| **Linguistics and Language Behavior Abstracts (LLBA) (ProQuest)** | S5 | S1 AND S2 AND S3 AND S4 |
|  | S4 | ti((experiment* or randomi?ed or randomly or RCT or trial or group* or control or placebo) ) OR ab((experiment* or randomi?ed or randomly or RCT or trial or group* or control or placebo) ) OR if((experiment* or randomi?ed or randomly or RCT or trial or group* or control or placebo) ) |
|  | S3 | (ti((Vocabulary or (Word knowledge or learn*)) or ((Linguistic or language or listening) and comprehen*) or (Language AND (proficien* or skill* or abilit* or acquisition)) or ((Oral or spoken) AND language) or Gramma* or Syntax or Syntactic* or Semantic* or Morph* or (Narrative AND (skill* or comprehen*)) or narration or (Figurative AND (speech or language or pragmatic*))) OR ab((Vocabulary or (Word knowledge or learn*)) or ((Linguistic or language or listening) and comprehen*) or (Language AND (proficien* or skill* or abilit* or acquisition)) or ((Oral or spoken) AND language) or Gramma* or Syntax or Syntactic* or Semantic* or Morph* or (Narrative AND (skill* or comprehen*)) or narration or (Figurative AND (speech or language or pragmatic*))) OR if((Vocabulary or (Word knowledge or learn*)) or ((Linguistic or language or listening) and comprehen*) or (Language AND (proficien* or skill* or abilit* or acquisition)) or ((Oral or spoken) AND language) or Gramma* or Syntax or Syntactic* or Semantic* or Morph* or (Narrative AND (skill* or comprehen*)) or narration or (Figurative AND (speech or language or pragmatic*)))) OR (MAINSUBJECT("Vocabulary") OR MAINSUBJECT("Word Formation") OR MAINSUBJECT("Linguistic Competence") OR MAINSUBJECT("Listening Comprehension") OR MAINSUBJECT("Language Proficiency") OR MAINSUBJECT("Language Acquisition") OR MAINSUBJECT("Oral Language") OR MAINSUBJECT("grammar") or MAINSUBJECT("syntax") or MAINSUBJECT("semantic") or MAINSUBJECT("morphology") or MAINSUBJECT("Narrative Structure") or MAINSUBJECT("narratives") or MAINSUBJECT("Figurative Language") ) |
|  | S2 | (ti((“language intervent*” or “language treat*” or “language educat*” or “language therap*” or “language train*” or “language facilit*” or “language program*” or “language acquisition”) ) OR ab((“language intervent*” or “language treat*” or “language educat*” or “language therap*” or “language train*” or “language facilit*” or “language program*” or “language acquisition”) ) OR if((“language intervent*” or “language treat*” or “language educat*” or “language therap*” or “language train*” or “language facilit*” or “language program*” or “language acquisition”) )) OR (MAINSUBJECT("Language Therapy") or MAINSUBJECT.EXPLODE("Language Acquisition") ) |
|  | S1 | (ti("Intellectual disabilit*" OR "Intellectual disorder*" OR "Developmental disorder*" OR "Developmental delay*" OR "Developmental disabilit*" OR "Mental retard*" OR "Language Disorder" OR "Specific Language Impair*" OR "SLI" OR "Developmental Language Disorder" OR "Language Delay*" OR "Language defic*" OR "Language impair*" OR "Language problem*" OR "Semantic‐pragmatic disorder*" OR "Expressive language disorder*" OR "Pervasive development* disorder*" OR "PDD" OR "PDD‐NOS" OR "Autis*" OR "Asperger" OR "ASD" OR "Down Syndrome" OR "Trisomy 21" OR "Chromosome 21" OR "Down* Disease") OR ab("Intellectual disabilit*" OR "Intellectual disorder*" OR "Developmental disorder*" OR "Developmental delay*" OR "Developmental disabilit*" OR "Mental retard*" OR "Language Disorder" OR "Specific Language Impair*" OR "SLI" OR "Developmental Language Disorder" OR "Language Delay*" OR "Language defic*" OR "Language impair*" OR "Language problem*" OR "Semantic‐pragmatic disorder*" OR "Expressive language disorder*" OR "Pervasive development* disorder*" OR "PDD" OR "PDD‐NOS" OR "Autis*" OR "Asperger" OR "ASD" OR "Down Syndrome" OR "Trisomy 21" OR "Chromosome 21" OR "Down* Disease") OR if("Intellectual disabilit*" OR "Intellectual disorder*" OR "Developmental disorder*" OR "Developmental delay*" OR "Developmental disabilit*" OR "Mental retard*" OR "Language Disorder" OR "Specific Language Impair*" OR "SLI" OR "Developmental Language Disorder" OR "Language Delay*" OR "Language defic*" OR "Language impair*" OR "Language problem*" OR "Semantic‐pragmatic disorder*" OR "Expressive language disorder*" OR "Pervasive development* disorder*" OR "PDD" OR "PDD‐NOS" OR "Autis*" OR "Asperger" OR "ASD" OR "Down Syndrome" OR "Trisomy 21" OR "Chromosome 21" OR "Down* Disease")) OR (MAINSUBJECT("Cognitive Development") OR MAINSUBJECT("Developmental Disabilities") OR MAINSUBJECT ("Mental Retardation") OR MAINSUBJECT.EXPLODE("Language Pathology") OR MAINSUBJECT.EXPLODE("Language Impairment") OR MAINSUBJECT("Asperger Syndrome") OR MAINSUBJECT("Autism") OR MAINSUBJECT ("Downs Syndrome") ) |
| **SpeechBite** |  | Intervention: Language therapy  Within population: Autistic spectrum disorders  Research Design: Randomised Controlled Trial  Intervention: Language therapy  Within population: Autistic spectrum disorders  Research Design: Non-Randomised Controlled Trial  Intervention: Language therapy  Within population: Intellectual disability  Research Design: Randomised Controlled Trial  Intervention: Language therapy  Within population: Intellectual disability  Research Design: Non-Randomised Controlled Trial  Intervention: Language therapy  Within population: Language Disorder (developmental)  Research Design: Randomised Controlled Trial  Intervention: Language therapy  Within population: Language Disorder (developmental)  Research Design: Non-Randomised Controlled Trial |
| **Latin American and Caribbean Health Sciences Literature (LILACS)** |  | (tw:(“Intellectual disabilit*” OR “Intellectual disorder*” OR “Developmental disorder*” OR “Developmental delay*” OR “Developmental disabilit*” OR “Mental retard*” OR “Language Disorder” OR “Specific Language Impair*” OR “SLI” OR “Developmental Language Disorder” OR “Language Delay*” OR “Language defic*” OR “Language impair*” OR “Language problem*” OR “Semantic‐pragmatic disorder*” OR “Expressive language disorder*” OR “Pervasive development* disorder*” OR “PDD” OR “PDD‐NOS” OR “Autis*” OR “Asperger” OR “ASD” OR “Down Syndrome” OR “Trisomy 21” OR “Chromosome 21” OR “Down* Disease”)) AND (tw:((“language intervent*” or “language treat*” or “language educat*” or “language therap*” or “language train*” or “language facilit*” or “language program*” or “language acquisition”) )) AND (tw:((experiment* or randomized or randomised or randomly or RCT or trial or group* or control or placebo) )) |
| **The Campbell Collaboration Library** |  | The search in this database was conducted from the Campbell Collaboration website. |
| **Google Scholar** |  | (Intellectual\|Developmental\|“Mental retard*”\|“Language Disorder”\|"language delay"\|down) “language therapy” (vocabulary\|word\|language\|linguistics\|listening\|narrative)  (experiment*\|randomized\|randomised\|randomly\|RCT\|trial\|  group*\|control\|placebo) |

# Online Supplement 2: Risk of Bias Assessment

| **Study** |  | **Selection bias** | | | | **Performance bias** | | **Detection bias** | | **Attrition bias** | | **Reporting bias** | |  |
| --- | --- | --- | --- | --- | --- | --- | --- | --- | --- | --- | --- | --- | --- | --- |
|  |  | **Q1. Sequence generation** | | **Q2. Allocation concealment** | | **Q3. Blinding of participants and personnel** | | **Q4. Blinding of outcome assessment** | | **Q5. Incomplete outcome data** | | **Q6. Selective outcome reporting** | |  |
| Adams et al. (2012) | Rating | Low risk of bias | | High risk of bias | | High risk of bias | | Low risk of bias | | Low risk of bias | | Unclear risk of bias | |  |
|  | Quotation | A researcher at the University of Manchester, who was not independent of the study, randomly assigned each child to SCIP intervention or treatment-as-usual (TAU) in a 2:1 ratio, stratified by age group (6;00–8;11 or 9;00–10;11). [...] Allocation used three possible permuted blocks of size three (SCIP TAU SCIP - SCIP SCIP TAU - TAU SCIP SCIP). A new block was selected after every three children in the same age band. | | [See quotation Q1] | | Families, schools and those delivering intervention could not be blind to treatment allocation | | All assessments (delivery, coding and scoring) were completed by a research assistant (RA) blind to treatment allocation. | | Attrition was therefore 3/88 (3%) for the primary endpoint. | | Not reported | |  |
| Baxter et al. (2022) | Rating | Low risk of bias | | Unclear risk of bias | | High risk of bias | | High risk of bias | | Low risk of bias | | Unclear risk of bias | |  |
|  | Quotation | Participants were allocated to the Intervention or Delayed Intervention group, using simple randomisation in Stata 17.0 (StataCorp, 2021) and all participants remained in the groups they were allocated to. | | Not reported | | The teaching assistants were not blind to whether they were in the intervention group [...] | | The assessor was not blind to the intervention group […] | | Data for one child are missing for t3 on all intervention measures in the Intervention Group and for one child on t3 TEGI probes in the Delayed Intervention group. | | Not reported | |  |
| Boyle et al. (2009) | Rating | Low risk of bias | | Low risk of bias | | Unclear risk of bias | | Low risk of bias | | Low risk of bias | | Low risk of bias | |  |
|  | Quotation | After informed, written parent and child consent was obtained, eligible children were allocated to mode stratified by city, using random number sequences generated by a statistical consultant via numerically sequenced, sealed envelopes opened by a project secretary. | | [See quotation Q1] | | Not reported | | All assessments post-baseline were carried out by SLTs not otherwise connected to the project to eliminate any bias resulting from the expectations of the research team. […] Language assessments post-intervention (T2) and 12 months post-intervention (T3) were carried out by qualified SLTs blind to therapy mode and not otherwise involved with the study. | | Accordingly, missing post-intervention scores for language outcomes were replaced by their pre-intervention baseline equivalents, or T2 scores where available, in the case of T3. [This is a conservative approach which minimizes any bias that can arise from participants withdrawing or dropping out of the study by assuming no change in children’s post-baseline scores]. There was missing data for eleven children in total. Nine were withdrawn by their parents after randomization, and a further two were withdrawn from the 12 months’ follow-up assessment having participated in the previous two assessments. | | The study is registered: International Standard Randomized Control Trial Number ISRCTN94684735 | |  |
| Burgoyne et al. (2012) | Rating | Unclear risk of bias | | Unclear risk of bias | | Unclear risk of bias | | Unclear risk of bias | | Low risk of bias | | High risk of bias | |  |
|  | Quotation | The 57 children (28 boys) recruited were randomly allocated to either the intervention or waiting control group. | | Not reported | | Not reported | | Children were assessed individually over two or more sessions on separate days. TAs were present during testing to assist with behavioural and communicative challenges where necessary. | | Four children withdrew from the intervention part way through the study (see Figure 1) but we obtained follow-up measures and included their scores in our analyses. […] In these analyses the small amount of missing data was dealt with using Full Information Maximum Likelihood (FIML) estimators (the default in Mplus). | | The trial was conducted within schools and hence was not registered. | |  |
| Calder et al. (2021) | Rating | Unclear risk of bias | | Low risk of bias | | Low risk of bias | | Low risk of bias | | Unclear risk of bias | | Unclear risk of bias | |  |
|  | Quotation | The children were assigned a code and assessed for eligibility. These codes were entered into a true random list generator by a researcher blinded to the purpose of the study to ensure concealed allocation sequence. [*Note:* low *N*] | | [See quotation Q1] | | The participants, their caregivers, and their teachers were not made aware of the conditions. The purpose of the intervention beyond targeting morphosyntax was not disclosed to caregivers or teachers. | | Assessors were blind to group assignment at all testing time points. | | However, one participant was exited from the study following crossover into the intervention condition due to reaching ceiling on pre-intervention measures and was deemed unlikely to benefit further from participation. Therefore, this participant was excluded from further analyses. One additional child in Group 2 was unavailable for testing at the final testing point due to being absent from the specialized educational program because of illness and was unavailable for follow-up. | | Not reported | |  |
| Casenhiser et al. (2013) | Rating | Low risk of bias | | Unclear risk of bias | | Unclear risk of bias | | Low risk of bias | | High risk of bias | | Unclear risk of bias | |  |
|  | Quotation | In each cohort, children were stratified by age and baseline level of language function, and were randomly assigned to one of two groups using random.org’s random number generator: 1) MEHRI treatment program (MEHRIT) or 2) Community Treatment (CT). | | Not reported | | Therapists were licensed speech-language pathologists or occupational therapists. | | All interactions were videotaped and later scored by a team of four coders who were blind to group assignment. [...] Licensed speech language pathologists who were unknown to the children and blind to group assignment conducted all speech assessments. | | This circumstance resulted in 9 families withdrawing from the MEHRIT treatment group and 13 withdrawing from the CT group. | | Not reported | |  |
| Crain-Thoreson & Dale (1999) | Rating | Unclear risk of bias | | Unclear risk of bias | | Unclear risk of bias | | Unclear risk of bias | | High risk of bias | | Unclear risk of bias | |  |
|  | Quotation | Based on the children’s pretest PPVT-R scores, triads of children with similar receptive vocabulary scores were formed. Children within each triad were then randomly assigned to the Parent (n = 13), Staff/practice (n = 13) or Staff/control (n = 11) group. (This procedure was followed within each of the three school districts participating in the study, leading to initially unequal numbers of participants in each group.) | | Not reported | | Not reported | | Each child completed the PPVT-R and the Expressive One-Word Vocabulary Test-Revised (EOWPVT-R; Gardner, 1990) at the beginning of the study. These measures were administered either by school personnel or by graduate student assistants trained in the administration of these measures. | | Thirty-seven children began the study but five did not complete it. | | Not reported | |  |
| Ebbels et al. (2012) | Rating | Unclear risk of bias | | Unclear risk of bias | | High risk of bias | | High risk of bias | | Low risk of bias | | Unclear risk of bias | |  |
|  | Quotation | The fifteen participants were randomly assigned to two groups (Therapy versus Waiting Control) by the first author using the random number function in Excel to sort the participants into a random sequence. The order of assignment of the random sequence to therapy group and phase had been predetermined (i.e. the first eight participants to the therapy group and the next seven to the waiting control group). [*Note:* low *N*] | | Not reported | | The therapy was usually provided by the participants’ own SLT as part of their normal therapy package. | | All post therapy testing was carried out by visiting speech and language therapy students who were blind to the participants’ group assignment. The majority of the pretherapy testing was carried out by a volunteer (a recently qualified SLT) who was also blind to group assignment. However, the volunteer was unable to complete the pre-therapy testing, so for five participants, pre-therapy testing on the TAWF and TWFD was carried out by their own SLT. | | No participant withdrew from the study at any point. However, two participants in the waiting control group did not complete the therapy programme in Phase 2. One (WC6) did not receive the last two 15-min sessions because he was unavailable due to other school activities. One (WC2) only received ten of the 16 sessions. | | Not reported | |  |
| Ebbels et al. (2014) | Rating | | Unclear risk of bias | | Unclear risk of bias | | High risk of bias | | Low risk of bias | | High risk of bias | | Unclear risk of bias | |
|  | Quotation | | The 14 participants were randomly assigned to two groups (Therapy versus Waiting Control) by the first author using the random number function in Excel to sort the participants into a random sequence which was then split into two groups. [*Note:* low *N*] | | Not reported | | The therapy was usually provided by the participants’ own SLT as part of their normal therapy package. | | This study is a single-blind cross-over trial with random allocation to groups with an assessor blind to group status. [...] All testing was carried out by an SLT assistant blind to group status, but trained in using the tests. | | Two participants did not receive the full 4 h of therapy. One (WC4) withdrew from the study during Phase 2 (his therapy phase). The other (T2) attended eight therapy sessions, but due to listening and attention difficulties had shorter sessions than the others, so in total received 2 h and 40 min of therapy. [...] Complete data were available for 12 participants, six in each group. | | Not reported | |
| Fey et al. (1993) | Rating | | Unclear risk of bias | | Unclear risk of bias | | Unclear risk of bias | | Low risk of bias | | Low risk of bias | | Unclear risk of bias | |
|  | Quotation | | These children were assigned randomly to one of three groups: the clinician treatment group, the parent treatment group, and the delayed-treatment group. Children identified during the first wave were assigned randomly to either the clinician treatment or the parent treatment group. After this initial group placement, the children were assigned randomly to receive treatment either immediately or after approximately a 41/2-month waiting period. | | Not reported | | Not reported | | At the time of the evaluations, the examiners were unaware of the group to which each child eventually was randomly assigned. | | The parents of the ninth child in the delayed-treatment group withdrew their child from the study because of unanticipated scheduling conflicts. | | Not reported | |
| Gallagher & Chiat (2009) | Rating | | Unclear risk of bias | | Unclear risk of bias | | Unclear risk of bias | | Low risk of bias | | Low risk of bias | | Unclear risk of bias | |
|  | Quotation | | In order to investigate the effectiveness of different therapy packages, children meeting the criteria for participation were randomly assigned to one of three intervention groups. […] The 24 participants were randomly assigned to the three intervention groups (n = eight per group). | | Not reported | | Not reported | | At the end of the intervention period, speech and language therapists from a different part of the service and blind to group status re-administered the assessments of comprehension of grammar and vocabulary as well as the expressive language tests. | | The majority of the children in both the Intensive and the Nursery-based groups attended all sessions. Two of the eight children in the Intensive group missed one session, and one child in the Nursery-based group missed two sessions. | | Not reported | |
| Gengoux et al. (2019) | Rating | | Low risk of bias | | Unclear risk of bias | | Unclear risk of bias | | Low risk of bias | | Unclear risk of bias | | Low risk of bias | |
|  | Quotation | | Eligible children were stratified on the basis of sex and were randomly assigned (1:1) to the treatment (PRT-P) or control (DTG) group via electronic generation of random numbers (www.randomizer.org) by a senior investigator not involved in the trial. | | Not reported | | Parent training was provided by master’s level clinicians who were supervised by the first author. In-home treatment was provided by bachelor’s-level clinicians who had demonstrated fidelity of implementation of PRT and who received weekly supervision. | | Consistent with previous research,^6,7^ raters blind to group assignment tallied the child’s total functional verbal utterances and also specified utterance type (i.e. unintelligible, imitative, verbally prompted, nonverbally prompted, or spontaneous). [...] Ratings were completed by raters blind to group assignment and time point | | Forty-eight subjects were randomly assigned (PRT-P group: n = 24; DTG: n = 24), and 43 families (PRT-P group: n = 23; DTG: n = 20) completed the 24-week trial. One participant in the PRT-P group withdrew when the family moved out of state; 4 participants in the DTG were excluded from final analyses after significant changes were made to concomitant therapies during the trial. | | This study was approved by Stanford University’s Institutional Review Board and was registered in the clinical trials database (clinicaltrials.gov; identifier NCT02037022). The full protocol is available on request. | |
| Green et al. (2010) | Rating | | Low risk of bias | | Low risk of bias | | High risk of bias | | Low risk of bias | | Low risk of bias | | Low risk of bias | |
|  | Quotation | | After consent was obtained and baseline assessments were done, the PACT manager allocated a sequential identification number and provided a statistician at the independent Christie Clinical Trials Unit in Manchester with the child’s number, treatment centre, age and autism severity. This statistician ran an allocation schedule that was computer-generated by use of probabilistic minimisation of imbalance in the marginal distribution of treatment centre, age (≤42 months or >42 months), and autism severity (ADOS-G). | | The statistician then telephoned the treatment allocation to the trial manager, who informed clinical sites. | | Assessors and supervising research staff were unaware of the treatment allocation; however, treatment allocation could not be masked from families and therapists. | | Assessors and supervising research staff were unaware of the treatment allocation [...]. Strict separation was kept between assessment and clinical data; assessors and therapists were located and supervised separately. Endpoint ADOS-G ratings were made from anonymised videotapes by an assessor from a different trial site to the ADOS-G administrator, unaware of the case details and treatment status. | | Multiple imputation, with the iterative-chained-equation method (ice procedure^25^), was used to complete the small amount of missing data. [...] Figure 1 shows the trial profile. The study over-recruited participants (final n = 152) compared with the target, and attrition to endpoint was low (6 [4%] of 152 for primary endpoint and 101 [7%] of 1520 for all secondary endpoints). | | This study is registered” International Standard Randomised Controlled Trial Number ISRCTN58133827 | |
| Haley et al. (2017) | Rating | | Low risk of bias | | Unclear risk of bias | | Unclear risk of bias | | High risk of bias | | Low risk of bias | | Unclear risk of bias | |
|  | Quotation | | The remaining children (*N* = 103, mean age 3;11) were then randomly allocated to either the oral language intervention or the waiting control group. The randomization was conducted using an algorithm in Excel created by one of the contributing authors, ensuring that the first author was initially blind to group membership. | | Not reported | | The programme was delivered by a teaching assistant (TA) selected by each nursery school. TAs delivering the programme were trained by the research team and received a detailed intervention manual. | | Testing was carried out by the first author and another psychology graduate student from the University of York, Psychology Department with assistance from trained psychology undergraduate students. All testers were blind to group membership with the exception of the first author who conducted on-site tutorials where she observed an intervention session taking place, thereby gaining awareness of group membership before post-testing occurred. | | CONSORT shows minimal loss to follow-up (1 treat; 4 wait list) [According to the flow chart, 5 out of 103 children dropped out.] | | Not reported | |
| Hardan et al. (2015) | Rating | | Low risk of bias | | Low risk of bias | | Unclear risk of bias | | Low risk of bias | | Low risk of bias | | Low risk of bias | |
|  | Quotation | | Randomization was done using a coin flip (www.random.org) by a senior investigator not involved in the clinical assessment or treatment. | | Sequence generation was done by the project coordinator who was not involved in the clinical ratings. | | Not reported | | The CGI ratings were assessed by a psychologist blind to group assignment and treatment phase, and were specifically focused on social and communication skills. | | Mixed effects regression models are advantageous compared to repeated measures ANOVA in that they accommodate missing time points, utilize all available data, and therefore can be considered truly intent-to-treat models. [...] Forty-seven subjects (PRTG = 25 [out of 27]; PEG = 22 [out of 26]) completed the study with one participant in the PEG group lost to follow-up. No adverse effects were noted in either group. | | This study was approved by Stanford University’s institutional review board and registered in the Clinical Trials database (NCT01881750; http://www.clinicaltrials.gov) | |
| Henry & Solari (2020) | Rating | | Unclear risk of bias | | Unclear risk of bias | | High risk of bias | | Unclear risk of bias | | Low risk of bias | | Unclear risk of bias | |
|  | Quotation | | This study used a matched pairs block design to randomly assign participating students to either the “business as usual” control condition or the experimental condition. Students were randomized across classrooms. […] First, students were ranked based on their performance on pretest measures of cognition, oral language, and comprehension. Second, the sample was split to create matched pairs based on these scores. Third, students in matched pairs were randomly assigned to an instructional group (experimental or control). | | Not reported | | Students’ special education teachers provided the intervention within their classrooms. The research team trained teachers during a one day, six-hour professional development session. | | Not reported | | Of the 44 students who participated in the intervention, one child was dropped from the study due to an extended absence from school. Data from 43 participants were included in the analyses. | | Not reported | |
| Hudson et al. (2017) | Rating | | Low risk of bias | | Unclear risk of bias | | Unclear risk of bias | | Unclear risk of bias | | Unclear risk of bias | | Unclear risk of bias | |
|  | Quotation | | For all three cohorts, the research methodologist randomized students (using only ID numbers) to one unique pair of conditions within the district using a random number generator in MS Excel (uniform distribution). | | Not reported | | Intervention sessions were provided by a total of 20 interventionists hired by the project from the local school community. | | All assessments were given by trained assessors familiar with special education, assessment procedures, and children with ASD. | | After attrition, which included student scheduling conflicts and moving from the district, there were 42, 46 and 45 students in Cohorts 1, 2 and 3, respectively, with attrition including 2 PA students in Cohort 2 and 6 PA students in Cohort 3. | | Not reported | |
| Joffe et al. (2019) | Rating | | Unclear risk of bias | | Unclear risk of bias | | High risk of bias | | Low risk of bias | | Low risk of bias | | Unclear risk of bias | |
|  | Quotation | | Students were randomized to the four groups within each school: (1) vocabulary intervention, (2) narrative intervention, (3) combined narrative and vocabulary intervention and (4) the delayed waiting control group. | | Not reported | | Each TA delivered all three interventions within their school. | | Assessments were conducted by the research team, who were blinded to group allocation. Those who assessed and enhanced fidelity of intervention, by monitoring and giving ongoing feedback, were not involved in assessments at those schools. [...] A total of 10% of participant responses at each assessment point were independently scored by a second coder, blind to group allocation. | | The rate of attrition from pre- to post-intervention was 5% and Little’s MCAR (Missing Completely at Random) test confirmed that missing data for all the language measures analyzed here could be considered to be missing completely at random (χ² = 185.79; d.f. = 177; p = .31). [...] Analyses were conducted in Stata 15.1 (Stata Corp, College Station, TX, USA) and structural equation models (SEM) were estimated in Mplus 8.0 (Muthen and Muthen, 1998–2017) with full information maximum likelihood estimators to allow for missing data and robust (Huber–White) standard errors to allow for the clustering of children within schools. | | Not reported | |
| Lavelli et al. (2019) | Rating | | High risk of bias | | High risk of bias | | Unclear risk of bias | | Low risk of bias | | Unclear risk of bias | | Unclear risk of bias | |
|  | Quotation | | Unfortunately, we could not use a group randomization because not all the families were able to commit to the intervention programme during the proposed period. Twenty families were therefore engaged in the intervention programme, while the other 12 families not able to participate acted as the comparison group. | | [See quotation Q1] | | Not reported | | Two research assistants who were blind to time and group coded all lines of transcriptions for mothers’ and children’s utterances, respectively. | | Not reported | | Not reported | |
| Lo & Shum (2021) | Rating | | Unclear risk of bias | | Unclear risk of bias | | Unclear risk of bias | | Low risk of bias | | Low risk of bias | | Unclear risk of bias | |
|  | Quotation | | Not reported | | Not reported | | Not reported | | All pretest and posttest assessments were conducted at the training centres by experimenters who were only involved in the assessments and not the intervention, and thus blinded to the group assignment of the children. | | There was no attrition from either group in this study. | | Not reported | |
| Lourenço et al (2020) | Rating | | High risk of bias | | High risk of bias | | Unclear risk of bias | | Unclear risk of bias | | Unclear risk of bias | | Unclear risk of bias | |
|  | Quotation | | It was not logistically possible and not ethical to conduct a randomized controlled trial in this case. All children were invited to participate in the narrative intervention programme. Not all parents (seven) accepted or could bring them to therapy. [*Note:* these children formed the control group.] | | [See quotation Q1] | | Not reported | | Eight weeks after the pretreatment assessment of the EG, the children were assess for post-treatment performance with the same assessment tasks that were used during the pretreatment phase. | | Not reported | | Not reported | |
| McDuffie et al. (2018) | Rating | | Unclear risk of bias | | Unclear risk of bias | | Unclear risk of bias | | Unclear risk of bias | | Unclear risk of bias | | Unclear risk of bias | |
|  | Quotation | | The 20 eligible boys and their mothers were randomly assigned after pretreatment assessment to either the active treatment group or treatment-as-usual comparison group using a list of random numbers. [*Note:* low *N*] | | Not reported | | Not reported | | Not reported | | Not reported | | Not reported | |
| Motsch & Marks (2015) | Rating | | Unclear risk of bias | | Unclear risk of bias | | Unclear risk of bias | | Low risk of bias | | Low risk of bias | | Unclear risk of bias | |
|  | Quotation | | The participating students were randomly assigned to two groups: a control group (CG, n = 79) and an experimental group (EG, n = 78). | | Not reported | | Not reported | | In order to guarantee objectivity, the post-tests were conducted blinded, such that the assessors did not know to which group each participant belonged. | | A total of 157 students with diagnosed lexical disorders participated in the study. At T2 and T3, four children could no longer be included in the tests because they had moved away or had a long term illness. These four children were excluded from all of the analyses reporting the degree of change between T1 and T3. Consequently, the final sample size after T3 is 153 children (EG: n = 76, CG: n = 77) | | Not reported | |
| Motsch & Ulrich (2012) | Rating | | Unclear risk of bias | | Unclear risk of bias | | Unclear risk of bias | | Unclear risk of bias | | Unclear risk of bias | | Unclear risk of bias | |
|  | Quotation | | The 54 children in the sample were randomly assigned to the two different groups: Children in the experimental group (EG) received the new strategy therapy ‘lexicon pirate’, in addition to the language support in kindergarten. Children in the control group (CG) were cared for exclusively in the kindergarten by a regular teacher and did not receive additional therapeutic intervention. | | Not reported | | Not reported | | To guarantee maximum objectivity of the results, the tests performed at T3 and T4 were ‘single-blinded’, meaning that the therapists performing the tests did not know which trial group the particular child belonged to. | | At the time of T4 (12 months after completion of the intervention), two children from the CG [out of 27] and one child from the EG [out of 27] could not be tested further as they had moved away from the area or they had a long-term disease. | | Not reported | |
| Pile et al. (2010) | Rating | | Low risk of bias | | Low risk of bias | | Unclear risk of bias | | Low risk of bias | | Unclear risk of bias | | Unclear risk of bias | |
|  | Quotation | | Once six children were recruited within a geographical location, the primary investigator, who was blind to the pre-test assessment results, used a random numbers table to assign them to experimental and control groups. | | Once six children were recruited within a geographical location, the primary investigator, who was blind to pre-test assessment results, used a random numbers table to assign them to experimental and control groups. | | Not reported | | Children in both groups were assessed at the pre-test and post-test by research assistants who were blind to the group assignment of the children. | | Not reported | | Not reported | |
| Rahman et al. (2016) | Rating | | Low risk of bias | | Low risk of bias | | High risk of bias | | Low risk of bias | | High risk of bias | | Low risk of bias | |
|  | Quotation | | [...] children were given a sequential identification number and randomly allocated via computer to the PASS (plus treatment as usual) group or treatment-as-usual alone group in a 1:1 ratio by an independent statistician at the Manchester Academic Health Sciences Clinical Trials Unit, UK [...]. Allocation was done with probabilistic minimisation, controlling for treatment centre (Goa or Rawalpindi), age (<6 years or ≥6 years), and functional impairment (VABS composite score <65 or ≥65). | | [See quotation Q1] | | Assessors and supervising research staff were masked to treatment allocation; however, treatment allocation could not be masked from families and therapists. | | Assessors and supervising research staff were unaware of the treatment allocation [...]. Strict separation was maintained between assessment and clinical data; assessors and therapists were located and supervised separately at both sites. To avoid the effects of familiarity, materials and location for assessment of children were different from those for the interventions. Assessors assessed baseline and endpoints using anonymised videotapes, unaware of the case details, assessment timepoint (i.e. baseline or endpoint), and treatment status. | | The linear regression models allowed analysis of all available data for primary and secondary outcomes without imputation, under the assumption that data were missing at random, conditional on the covariates [...]. Six (9%) of 65 participants were lost to follow up. [...] 26 (81%) of 32 participants in the intervention group completed the 12 sessions of treatment. | | Not reported | |
| Roberts & Kaiser (2012) | Rating | | Unclear risk of bias | | Unclear risk of bias | | Unclear risk of bias | | Unclear risk of bias | | Unclear risk of bias | | Unclear risk of bias | |
|  | Quotation | | Children with LI were randomly assigned to the LI-treatment or the LI-control group (i.e. business as-usual). [...] After the initial screening, eligible children with LI were randomly assigned to the treatment or control group. | | Not reported | | Not reported | | Not reported | | Not reported | | Not reported | |
| Roux et al. (2015) | Rating | | Unclear risk of bias | | Unclear risk of bias | | Unclear risk of bias | | Unclear risk of bias | | Low risk of bias | | Unclear risk of bias | |
|  | Quotation | | A randomized block design was used to assign the groups of students to the control and intervention conditions. In each school, participating students with similar oral (PPVT) and reading (word recognition) skills were assigned to groups of two to four members. [...] Within each block, one group was randomly assigned to the control condition and the other to the intervention condition (see Tables 1 and 2 for condition equivalence). | | Not reported | | Not reported | | To avoid introducing a bias, the assistant assessing the student at post-test or follow-up was not the one who offered the intervention to this student. | | *See Figure 1:* 43 [intervention: 23 out of 24, control group: 20 out of 21] children completed the study. 4 children dropped before the follow-up. | | Not reported | |
| Sajaniemi et al. (2010) | Rating | | Unclear risk of bias | | Unclear risk of bias | | Unclear risk of bias | | Low risk of bias | | Unclear risk of bias | | Unclear risk of bias | |
|  | Citation | | Not reported | | Not reported | | Not reported | | The examiner was blinded for the allocation of the children into intervention and control groups. | | Not reported | | Not reported | |
| Salt et al. (2002) | Rating | | High risk of bias | | High risk of bias | | Unclear risk of bias | | Unclear risk of bias | | High risk of bias | | Unclear risk of bias | |
|  | Quotation | | Random allocation to groups was not feasible as it would not be ethically acceptable to withhold suitable treatment, i.e. some children would have missed their opportunity for a preschool treatment. | | [See quotation Q1] | | Not reported | | Not reported | | Three participants dropped out. | | Not reported | |
| Sepúlveda et al. (2013) | Rating | | Unclear risk of bias | | Unclear risk of bias | | High risk of bias | | Unclear risk of bias | | Unclear risk of bias | | Unclear risk of bias | |
|  | Quotation | | Of these individuals, ten were assigned randomly to the experimental group (four girls and six boys) and the other ten to the control group (five girls and five boys). | | Not reported | | These therapists were the children’s habitual therapists. | | Not reported | | Not reported | | Not reported | |
| Sokmum et al. (2017) | Rating | | High risk of bias | | High risk of bias | | Unclear risk of bias | | Unclear risk of bias | | Unclear risk of bias | | Unclear risk of bias | |
|  | Quotation | | The limitation of the present study is the lack of random assignment of participants to the intervention and control group. However, both groups were paired according to the gender, stage of child’s communication, language score and parent’s education level. | | [See quotation Q1] | | Not reported | | Not reported | | Not reported | | Not reported | |
| Solari et al. (2020) | Rating | | Unclear risk of bias | | Unclear risk of bias | | Unclear risk of bias | | Low risk of bias | | Unclear risk of bias | | Unclear risk of bias | |
|  | Quotation | | Not reported | | Not reported | | Not reported | | Two graduate student researchers, who were blind to group status and child identity, independently coded all narratives. | | Not reported | | Not reported | |
| Solomon et al. (2014) | Rating | | Low risk of bias | | Unclear risk of bias | | Unclear risk of bias | | Low risk of bias | | Low risk of bias | | Low risk of bias | |
|  | Quotation | | Deidentified demographic and diagnostic data for enrolled families were sent to the MSU research team for randomization to usual community services (CS) intervention or PLAY plus CS. Randomization was computer-generated and occurred within sites using a matched pair design with primary blocking variables [...] | | Not reported | | 6 PLAY consultants (1 occupational therapist, 2 speech and language therapists, and 3 special educators) were employed at the 5 ES study sites and had 2 to 5 years of experience as PLAY consultants. All had been trained to certification by attending a 4-day training seminar followed by 12 to 18 months of supervision. | | The ADOS and Mullen were administered in ES offices, and evaluators were blinded as to intervention status. | | Apart from the Mullen and MCDI-WS, missing data were missing completely at random (Little’s MCAR test x2 [42, N 5 112] 5 50.16, p 5 .181). Data were imputed forward for using the last observation carried forward method at the scale level. Although multiple imputation procedures are promising,^49^ the field has not advanced to provide methods for addressing repeated measures ANOVA analysis,^50^ as used in this article. A common alternative has been the use of last observation car ried.^51,52^ Analyses used an intent-to-treat approach, which included all cases randomized to PLAY and CS groups including those that did not complete the intervention. Exceptions were the video-based MBRS, CBRS, and FEAS, which were rated only for cases that completed the intervention. | | Clinical Trials Registry Number NCT01768806 | |
| Starling et al. (2012) | Rating | | High risk of bias | | Low risk of bias | | High risk of bias | | Low risk of bias | | Unclear risk of bias | | Unclear risk of bias | |
|  | Quotation | | We approached an administrator of government schools, independent of the current study, who located a group of demographically similar secondary schools in metropolitan Sydney, Australia. A short list of schools was identified by the administrator, and two secondary schools agreed to participate in the study. [*Note:* only two schools] | | A concealed randomization process occurred following identification of a cohort of teacher and student participants across the two schools. Whole schools were randomly assigned to the treatment or control condition by means of sealed envelopes chosen by a person independent of the study. | | The first author, an experienced and certified SLP, administered the training program. | | All assessments, scorings, and ratings were carried out by research assistants (RAs) who were blinded to the nature of the intervention, the trained/control condition of the cohorts, and the test phases of the study. | | Not reported | | Not reported | |
| van der Schuit et al. (2011) | Rating | | Unclear risk of bias | | Unclear risk of bias | | Unclear risk of bias | | Unclear risk of bias | | Unclear risk of bias | | Unclear risk of bias | |
|  | Quotation | | Not reported | | Not reported | | Not reported | | Not reported | | Not reported | | Not reported | |
| van Kleeck et al. (2006) | Rating | | High risk of bias | | Unclear risk of bias | | Unclear risk of bias | | High risk of bias | | Unclear risk of bias | | Unclear risk of bias | |
|  | Quotation | | To ensure random assignment, the children were assigned alternatively to either the treatment or the control group as the children entered the study when their guardians returned the permission forms. | | Not reported | | Children in the treatment group participated in individual 15/min book/sharing at their Head Start programs twice per week for 8 weeks with trained graduate and undergraduate research assistants from programs in communication sciences and disorders. | | Unfortunately, most of the testers were not blind to the children's group (treatment or control) status. | | Not reported | | Not reported | |
| Wake et al. (2013, 2015) | Rating | | Low risk of bias | | Low risk of bias | | High risk of bias | | Low risk of bias | | Unclear risk of bias | | Low risk of bias | |
|  | Quotation | | Eligible children were allocated to intervention or “usual care” (control) arms in a 1:1 ratio by an independent researcher by using a computer generated random number sequence, concealed using sealed opaque envelopes. Randomization was stratified by previous trial (Let’s Read or Let’s Learn Language) and nature of language problem (receptive, expressive, or both receptive and expressive), and blocked within each stratum using randomly permuted block sizes in a nonsystematic sequence. | | [see quotation Q1] | | [...] once allocated, participants could not be blinded. | | Outcome assessors were blind to group allocation [...] | | Ninety-one intervention (92% of 99) and 88 control (87% of 101) children were retained at age 5 years. Of the 99 intervention families, 94%, 91%, and 85% received at least 1, 2, and 3 blocks of therapy, respectively; on average, families received 17 of the 18 intervention sessions. | | This trial has been registered with the ISRCTN Register (http://isrctn.org) (identifier ISRCTN03981121). | |
| Washington et al. (2011) | Rating | | High risk of bias | | High risk of bias | | High risk of bias | | Low risk of bias | | Low risk of bias | | Unclear risk of bias | |
|  | Quotation | | Following parental consent, participants were consecutively assigned to C-AT or nC AT and subsequently completed testing related to the study. [...] To establish the impact of C-AT or nC-AT over NT, a control group was required. Parents of children who were not receiving treatment (i.e. awaiting treatment) were asked to participate. The types of morphological errors demonstrated by these children were consistent with treatment participants. This convenience sample of children served as control participants, no treatment (NT). | | [See quotation Q1] | | The first author provided all intervention sessions. | | Administration of the SPELT-P was completed pre-, post- and 3-months post-treatment by blinded assessors. Individuals trained to collect and transcribe language samples were partially blinded to assessment time point. | | Participants were between the ages of 3;6 and 4;11 (years; months, M = 4;3) at the outset of treatment and met the inclusion criteria with no attrition. | | Not reported | |
| Westerveld et al. (2021) | Rating | | Unclear risk of bias | | Unclear risk of bias | | Unclear risk of bias | | Unclear risk of bias | | High risk of bias | | Unclear risk of bias | |
|  | Quotation | | Children were randomly allocated, using SPSS, to the intervention or waitlist control group. | | Not reported | | Not reported | | Not reported | | *See Figure 1:* 11 children dropped the study [intervention: 3 out of 12, control group: 8 out of 11]. As shown in Figure 1, 25% of the intervention group withdrew due to other commitments/parental workload. In the waitlist control group, 4 parents withdrew and reported timing issues or workload by the time the intervention was offered. [...] Missing values analysis including all variables showed < 5% missing with analysis, suggesting data were missing completely at random, Little’s MCAR test χ2 (29) 25.81, *p* = .636. | | Not reported | |
| Wright et al. (1993) | Rating | | Unclear risk of bias | | Unclear risk of bias | | Unclear risk of bias | | Unclear risk of bias | | Unclear risk of bias | | Unclear risk of bias | |
|  | Quotation | | Fifteen of the children, 14 boys and one girl, were assigned to the experimental groups; the other 15 children, 11 boys and four girls acted as the controls. | | Not reported | | Not reported | | These were administered by an SLT other than one who had taken part in the intervention therapy with the subject. | | Eight were allocated to the group which was to receive semantic therapy, and seven to the phonological therapy group. The latter group was reduced to six when one of the pupils was hospitalized during the course of the programme. | | Not reported | |

# Online Supplement: Figures

**Figure S1**. Sensitivity analysis via the leave-one-out method. The line with dots represents the variation of the overall meta-analytic mean effect size as each single study is removed from the overall model; the shaded area represents the variation of its 95% CI.


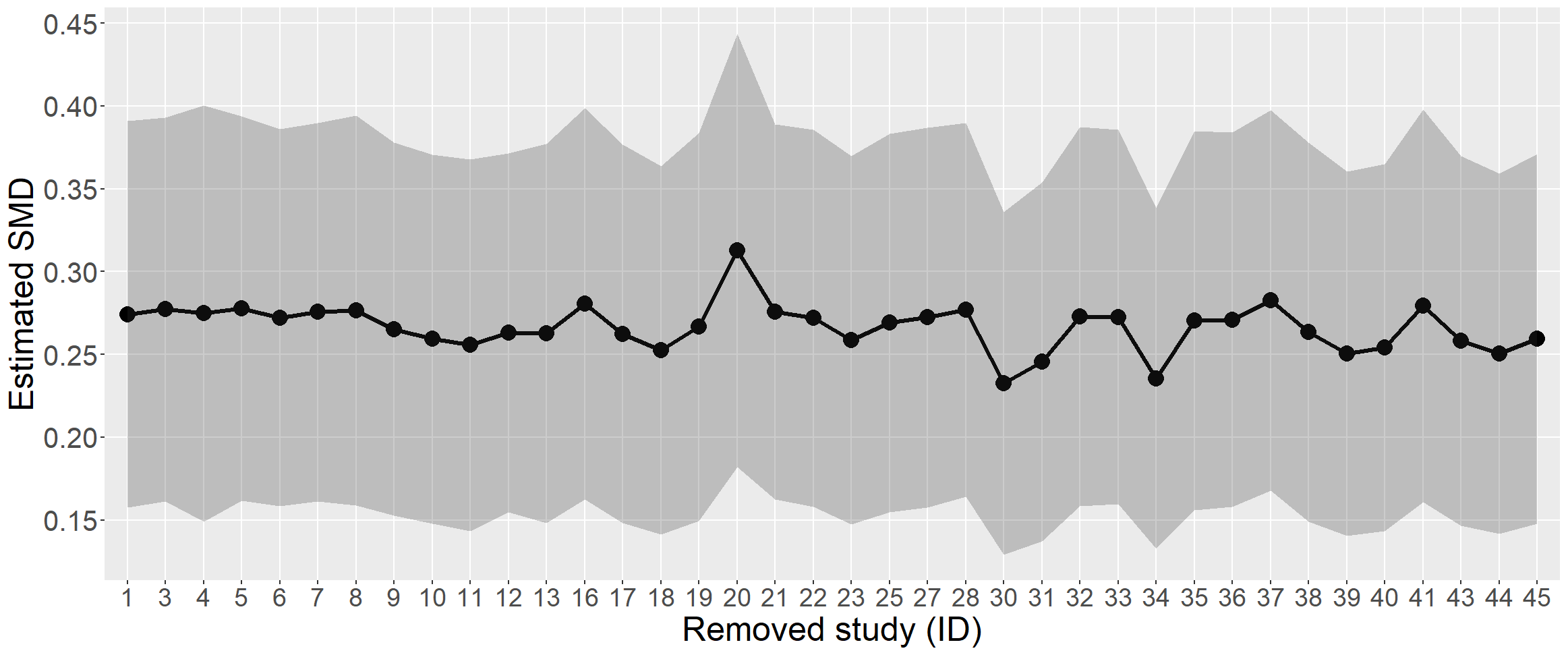


**Figure S2.** Forest plot of all effects for pretest-follow-up comparisons aggregated by comparison-within-study. Error bars represent 95% CIs. Larger dots indicate higher precision (i.e. smaller standard errors). The diamond below represents the overall meta-analytic mean effect (its width represents the 95% CI).

**
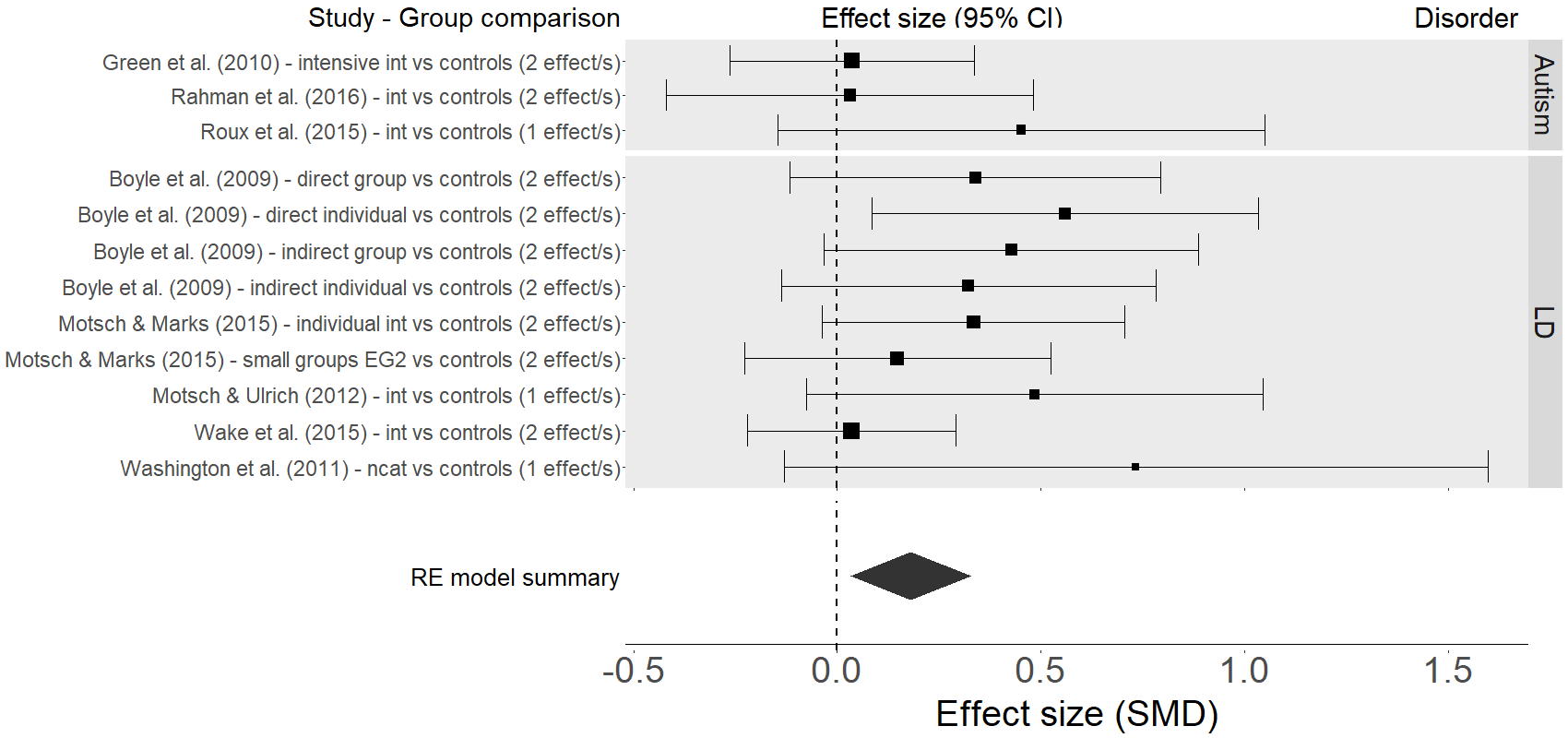
**

**Figure S3.** Funnel plot of all effects of pretest-follow-up comparisons (graphically clustered by study) with a PET meta-regression slope and PET-corrected estimate (dot with 95% CI at SE = 0).


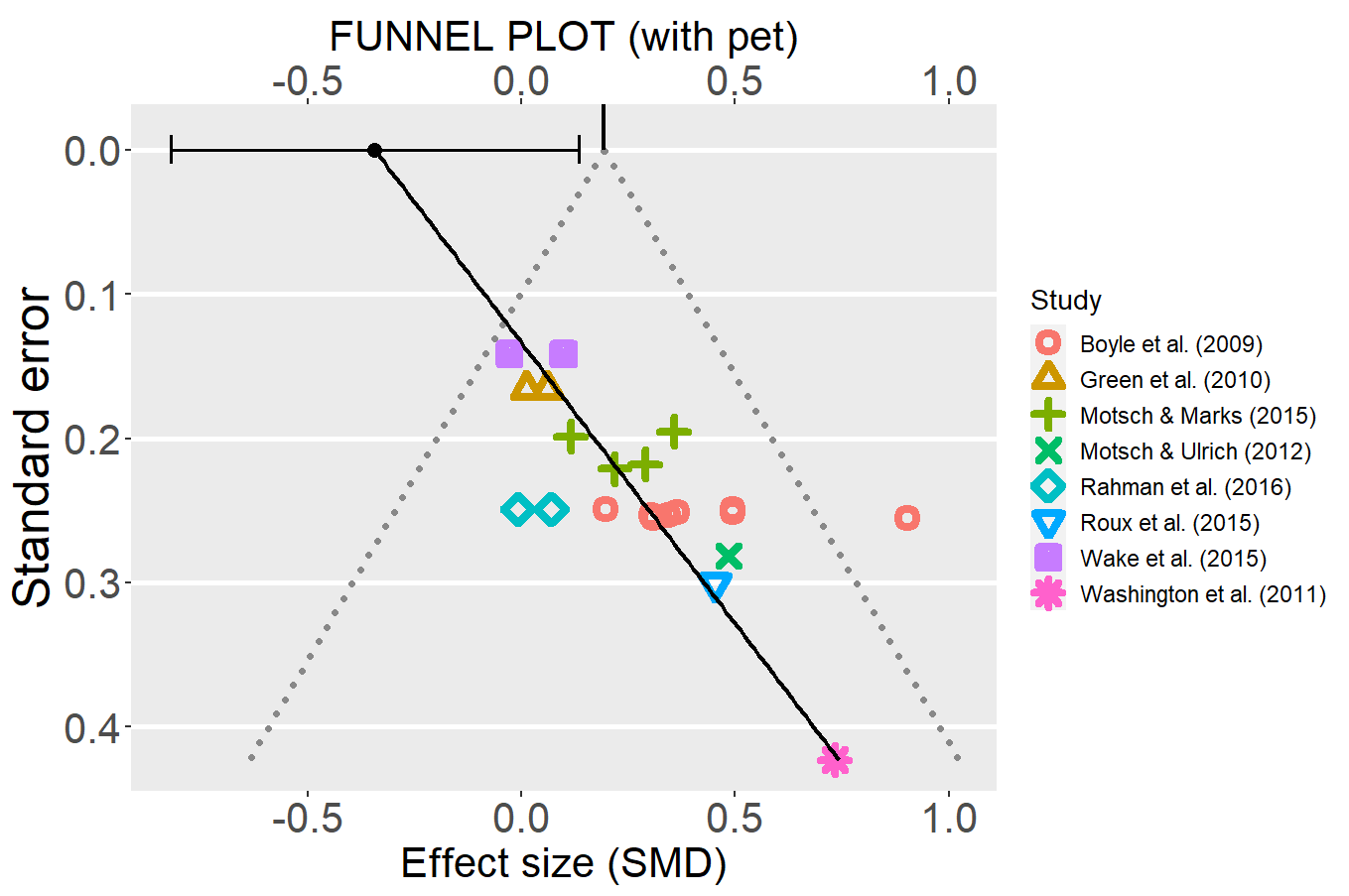

Supplement: Supplementary file 1 — Supporting information. [file CL2-19-e1368-s001.docx]
